# Extracellular CahB1 from *Sodalinema gerasimenkoae* IPPAS B-353 Acts as a Functional Carboxysomal β-Carbonic Anhydrase in *Synechocystis* sp. PCC6803

**DOI:** 10.3390/plants12020265

**Published:** 2023-01-06

**Authors:** Jun Minagawa, Marcel Dann

**Affiliations:** 1 Division of Environmental Photobiology, National Institute for Basic Biology (NIBB), Aichi, Okazaki 444-8585, Japan; 2 Plant Molecular Biology, Ludwig-Maximilian University (LMU) Munich, 82152 Planegg, Germany

**Keywords:** cyanobacteria, carbonic anhydrase, carboxysome, *Synechocystis*, *Microcoleus chthonoplastes*, *Coleofasciculus chthonoplastes*, *Sodalinema gerasimenkoae*, protein secretion

## Abstract

Cyanobacteria mostly rely on the active uptake of hydrated CO_2_ (i.e., bicarbonate ions) from the surrounding media to fuel their inorganic carbon assimilation. The dehydration of bicarbonate in close vicinity of RuBisCO is achieved through the activity of carboxysomal carbonic anhydrase (CA) enzymes. Simultaneously, many cyanobacterial genomes encode extracellular α- and β-class CAs (EcaA, EcaB) whose exact physiological role remains largely unknown. To date, the CahB1 enzyme of *Sodalinema gerasimenkoae* (formerly *Microcoleus*/*Coleofasciculus chthonoplastes*) remains the sole described active extracellular β-CA in cyanobacteria, but its molecular features strongly suggest it to be a carboxysomal rather than a secreted protein. Upon expression of CahB1 in *Synechocystis* sp. PCC6803, we found that its expression complemented the loss of endogenous CcaA. Moreover, CahB1 was found to localize to a carboxysome-harboring and CA-active cell fraction. Our data suggest that CahB1 retains all crucial properties of a cellular carboxysomal CA and that the secretion mechanism and/or the machinations of the *Sodalinema gerasimenkoae* carboxysome are different from those of *Synechocystis*.

## 1. Introduction

Carbonic anhydrases (CAs; EC 4.2.1.1) form a polyphyletic group of enzymes currently comprising eight classes (α, β, γ, δ, ζ, θ, η, ι; [1]) and are ubiquitous throughout the tree of life. Catalysing the bi-directional interconversion of water-dissolved carbon dioxide (CO_2_/H_2_O) and bicarbonate (HCO_3_^−^/H^+^), CAs are integral to carbon-based metabolism, especially by facilitating photosynthetic carbon fixation through release of CO_2_ in the vicinity of RuBisCO in the context of cyanobacterial and also microalgal carbon-concentrating mechanisms (e.g., [2,3,4,5]). Moreover, when acting outside the cell, CAs are presumed to facilitate the uptake of inorganic carbon from the surrounding media as bicarbonate [6]. In the model cyanobacterium *Synechocystis* sp. PCC6803 (hereafter *Synechocystis*), the β-CA CcaA (Slr1347) and the γ-CA-like protein CcmM (Sll1031) constitute crucial components of a RuBisCO-associated bicarbonate dehydration complex within its β-type carboxysome [7]. While CcmM has been shown to be structurally important to bicarbonate dehydration complex organization [7], the enzymatic function of CcmM as an active CA has been lost in *Synechocystis* and various other beta-cyanobacteria [8,9]. *Synechocystis* CcaA, meanwhile, has been confirmed to be an active CA enzyme [10,11] which is constitutively expressed under a range of exogenous CO_2_ supply conditions [12,13,14,15]. Under atmospheric CO_2_ concentrations, CcaA has been shown to be indispensable for photoautotrophic growth (i.e., the *ccaA* knockout allele was found impossible to fully segregate), while elevated CO_2_ (5% *v/v*) allows for full knockout segregation [16]. For β-cyanobacteria, CcaA is hypothesized to be a later acquisition via horizontal gene transfer, while CcmM is seemingly an ancestral protein which likely maintained its catalytic activity in a range of β-cyanobacteria, some of which did and some of which did not acquire CcaA [8].

Besides carboxysomal CAs, many cyanobacterial genomes also encode α- and β-CAs with predicted extracellular localization [17]. Despite their phylogenetic abundance and presumed role in inorganic carbon assimilation, few such enzymes have so far been shown to accumulate outside the cell of cyanobacteria and to be enzymatically active.

An extracellular α-CA (EcaA) from *Synechococcus* sp. PCC7942 (hereafter *Synechococcus*) has been described to accumulate under elevated CO_2_ concentrations [18], and although its physiological role remains elusive, its recombinant protein has recently been found to possess catalytic activity [19]. Similarly, EcaA from *Crocosphaera subtropica* (formerly *Cyanothece*) sp. ATCC 51142 has been found to be an active enzyme [20] possessing a signal peptide that efficiently elicits protein secretion in *Escherichia coli* [21].

For β-class CAs, records of active and secreted enzymes are scarce and contradictory. The *Synechocystis* genome encodes a presumably extracellular β-CA (EcaB; Slr0051) which has been found to localize to the periplasm in proteomics studies [22] and to be enzymatically inactive [11], while a more recent report proposes EcaB to be a thylakoid-localized and active enzyme [23]. In a second instance, the haloalkaliphilic and biofilm-forming *Sodalinema gerasimenkoae* (formerly *Microcoleus*/*Coleofasciculus chthonoplastes*) has been found to possess active and extracellular CAs [24,25], and the β-CA CahB1 has been shown to localize exclusively to the outer-membrane fraction of the cell [26]. Moreover, enzymatic CA activity of recombinant CahB1 protein [26] and its susceptibility to standard CA inhibitors, such as acetazolamide [27], could be biochemically confirmed, and recently, recombinant CahB1 was also reported to be employed to foster biotechnological calcium carbonate precipitation [28]. This is well in line with its proposed role in stromatolite formation [26], and a likely side effect of extracellular CA activity fostering efficient dissolved inorganic carbon uptake in alkaliphilic phototrophic biofilms [17].

In vivo, however, the CahB1 protein represents a veritable conundrum. While CahB1 has been shown to localize to the extracellular matrix outside the outer membrane, it is absent from the carboxysome [26]. Still, CahB1 shows strong overall similarity to the typically carboxysome-localized β-CA CcaA rather than to other β-class EcaB enzymes that are predicted to be localized outside of cells, sharing about 60 percent sequence identity and about 80 percent sequence similarity with *Synechocystis* CcaA [26], thus exceeding the highest reported pairwise identity values of ~55 percent for *Synechocystis*/*Synechococcus* CcaAs and/or higher plant chloroplast β-CAs [29]. Furthermore, CahB1 lacks a predicted secretion-signal peptide at its N-terminus [26], and recombinant CahB1 protein was not secreted at all by its *Escherichia coli* expression host [21,26], raising questions about its mode of translocation into the pericellular space or beyond the outer membrane. Moreover, the *Sodalinema gerasimenkoae* IPPAS B-353 (hereafter *Sodalinema gerasimenkoae*) genome sequence [30], previously reported as *Microcoleus chthonoplastes* IPPAS B-353 (synonymous IPPAS B-270) genome [31], does not encode any other CcaA homologues besides CahB1 [31]. This is in sharp contrast to the vital importance of *Synechocystis* and *Synechococcus* CcaA to the function of their carboxysomes [16,32] and begs the question of the identity of the *Sodalinema gerasimenkoae* carboxysomal CA.

To assess whether in cyanobacteria CahB1 intrinsically localizes outside the cell, or whether it actually acts as a carboxysomal β-CA, we employed *Synechocystis* as a heterologous test system to study the effects of heterologous CahB1 expression in the presence and absence of endogenous CcaA. CahB1 was found to enhance photoautotrophic growth when added to the endogenous system. The CahB1 protein was found to accumulate within the cell in a carboxysome-harboring cell fraction and to functionally complement *ccaA* knockout mutants. Our data hence suggests that within the *Synechocystis* cell CahB1 can assume the role of a functional β-carboxysomal CA and even stimulate carboxysomal CA activity when being co-expressed with endogenous CcaA.

## 2. Results

### 2.1. CahB1 Is Highly Homologous to Synechocystis CcaA

**Figure 1:**
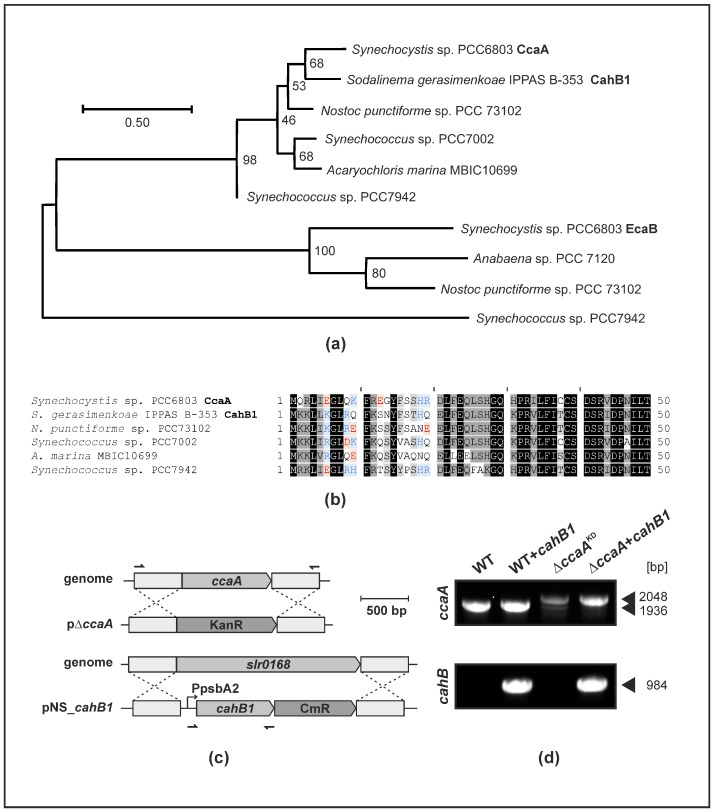
*Sodalinema gerasimenkoae* CahB1 is highly homologous to *Synechocystis* CcaA. (**a**) Maximum likelihood (log likelihood −2438.93) phylogenetic tree reconstruction of β-CA from different representative model cyanobacteria. Numbers next to nodes indicate bootstrap support over 200 replicates. The branch lengths are proportional to the number of substitutions per site (see scale bar). The analysis involved 10 amino acid sequences with a total of 174 homologous positions in the final dataset. (**b**) N-terminal protein sequence alignment of CcaA/CahB1 homologues included in (**a**). Conserved positions (black), highly similar positions (>80% similarity/identity; dark grey), and similar positions (>60% similarity/identity; light grey) are highlighted. Non-conserved charged amino acid residues in the first 20 positions are highlighted in blue (basic) and red (acidic). (**c**) Schematic maps of the pΔ*ccaA* knock-out and the pNS_*cahB1* knock-in constructs used in this study. KanR, kanamycin resistance gene *nptI*; CmR, chloramphenicol resistance gene *cat*; PpsbA2, promoter of *Synechocystis psbA2* gene encoding D1 subunit of photosystem II; bp, base pairs. Upstream/downstream regions used for homologous recombination with genomic target loci *ccaA* (*slr1347*) and neutral site (*slr0168*) are indicated as grey boxes. Primer-binding sites for genotyping PCR are indicated as half arrows. (**d**) Genotyping PCR of *Synechocystis* wildtype (*WT*), wildtype transformed with a CahB1 expression construct (WT + *cahB1*), mutant harbouring the *ccaA* deletion construct (Δ*ccaA*^KD^), and mutant harbouring the *ccaA* deletion construct transformed with *cahB1* expression construct (Δ*ccaA + cahB1*). Positions and sizes of the WT *ccaA* locus amplicon (1936 bp), the Δ*ccaA* locus amplicon (2048 bp), and the *cahB1* expression construct amplicon (984 bp; PpsbA2:*cahB1*) are highlighted with arrowheads. Note the change from incomplete to complete segregation status of Δ*ccaA* upon introduction of the *cahB1* gene.

A close similarity of primary sequences and their hydrophobicity profiles has been described for CahB1 and *Synechocystis* CcaA [26]. According to maximum likelihood phylogenetic analyses, the CahB1 protein of *Sodalinema gerasimenkoae* clusters with CcaA homologues of other β-cyanobacterial model species rather than with the β-CA EcaB proteins that are predicted to be localized outside of cells (Figure 1a). The 50 amino acid residues comprising the N-termini of CcaA/CahB1 are highly conserved, with the exception of CahB1 lacking any negatively charged amino acid residues in its first 20 positions (Figure 1b). Meanwhile, none of the investigated CcaA/CahB1 homologues are predicted to contain a secretion signal peptide (see Supplementary Materials File S1).

To assess the functionality of CahB1 within *Synechocystis*, the ORF encoding CahB1 was cloned into an expression construct targeting the designated genomic neutral site *slr0168*. CahB1 was expressed under control of the strong *psbA2* promoter and co-introduced with a chloramphenicol resistance gene to enable positive selection (Figure 1c). To generate knockout strains of the endogenous *ccaA* gene for complementation studies, the *slr1347* ORF was targeted by homologous recombination and replaced with a kanamycin resistance gene (Figure 1c). Upon introduction into the *ccaA*-depleted mutants, the *cahB1* expression construct was found to allow for segregation of the Δ*ccaA* knockout allele under atmospheric CO_2_ while transformants harbouring only the *ccaA* KO construct do not segregate completely under the same conditions (resulting in effective knock-down of *ccaA*; hereafter Δ*ccaA*^KD^) (Figure 1d). This indicates that some copies of the endogenous *ccaA* gene are indispensable under atmospheric CO_2_ unless its function is complemented by a foreign gene, such as *cahB1*. This result is compatible with the previous reports [16,32].

The close resemblance of CahB1 to CcaA, as well as the capacity of *cahB1* to genetically complement Δ*ccaA*, are highly suggestive of a role of CahB1 as carboxysomal β-CA rather than as a secreted β-CA in *Synechocystis*.

### 2.2. CahB1 Functionally Complements Loss of Synechocystis CcaA

**Figure 2:**
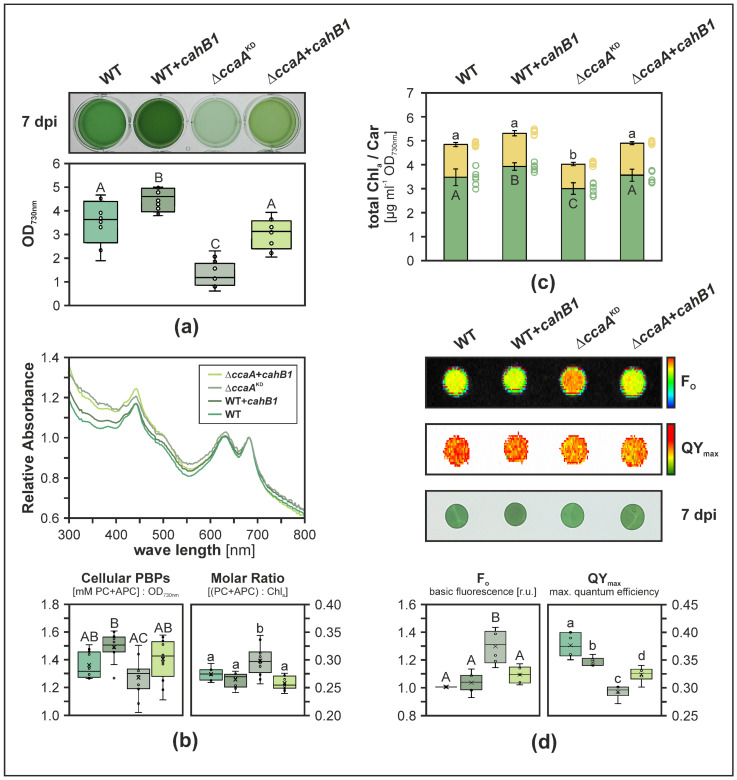
CahB1 complements loss of *Synechocystis* CcaA. (**a**) Representative photoautotrophic culture phenotypes. dpi, days past inoculation. Boxplots represent data from *n* = 12 biological replicates. (**b**) Average whole cell UV/Vis absorbance spectra normalized to the chlorophyll *a* absorbance maximum at λ = 683 nm (top), and derived estimates of total cellular phycobiliproteins (PBPs) normalized to OD_730nm_ (bottom left) and molar PBPs-to-chlorophyll *a* (Chl_a_) ratios (bottom right). PC, phycocyanin; APC, allophycocyanin; *n* = 12 biological replicates. (**c**) Cellular chlorophyll *a* and carotenoid (Car) contents of OD_730nm_ = 0.75 cell equivalents. Horizontal lines show average values, error bars show sample standard deviations, and dots show individual replicate values. *n* = 6 biological replicates. (**d**) Photosynthetic performance parameters F_o_ (minimal fluorescence relative to WT) and QY_max_ (photosystem II maximum quantum yield) of 1 mL OD_730nm_ = 0.75 cell equivalents harvested 7 dpi dropped onto BG11 agar. F_o_ normalized to corresponding WT samples. *n* = 6 biological replicates. Letters indicate statistically significant differences (*p* ≤ 0.05) according to multiple simultaneous comparisons in post hoc Bonferroni–Holm-corrected Tukey HSD (honest significant difference) tests after statistically significant among-group differences were detected by one-way ANOVA ((**a**) *p* = 4.13 × 10^−14^; (**b**) *p* = 9.00 × 10^−4^ [cellular PBPs] and *p* = 2.48 × 10^−4^ [molar ratio PBPs:Chl_a_]; (**c**) *p* = 7.18 × 10^−5^ [Chl_a_] and *p* = 8.27 × 10^−7^ [Car]; (**d**) *p* = 1.06 × 10^−5^ [F_o_] and *p* = 4.56 × 10^−8^ [QY_max_]).

Strains expressing the *cahB1* gene in the WT background (i.e., harbouring both *Synechocystis* CcaA and CahB1; WT + *cahB1*) did grow to significantly higher final optical densities than parental WT (*p* = 2.3 × 10^−3^), while Δ*ccaA*^KD^ strains were significantly impaired in growth (*p* = 6.6 × 10^−10^). This growth defect was alleviated in Δ*ccaA* + *cahB1* strains (Figure 2a), which was in line with our genetic complementation data (Figure 1d). Based on whole-cell absorbance-spectra-derived estimates for all strains, cellular phycobiliprotein (PBP) contents were found largely unaltered with Δ*ccaA* + *cahB1* levels being restored to WT levels, while WT + *cahB1* displayed sightly increased (*p* = 5.4 × 10^−2^) and Δ*ccaA*^KD^ significantly decreased (*p* = 4.5 × 10^−4^) cellular PBP levels, respectively (Figure 2b). Correspondingly, the molar ratio of PBPs (phycocyanin and allophycocyanin) to chlorophyll *a* was significantly increased in Δ*ccaA*^KD^ (Figure 2b; *p* = 4.0 × 10^−3^). These findings parallel significant increases and reductions in cellular chlorophyll *a* content in WT + *cahB1* (*p* = 2.3 × 10^−2^) and Δ*ccaA*^KD^ (*p* = 2.0 × 10^−2^), respectively, while total cellular carotenoids were significantly reduced only in Δ*ccaA*^KD^ mutants (*p* = 5.4 × 10^−6^). Both chlorophyll *a* and carotenoid contents were recovered in Δ*ccaA* + *cahB1* (Figure 2c). These findings indicate proportionate increases in cellular PBPs and chlorophyll *a* levels in WT + *cahB1*, and slightly stronger reductions in cellular chlorophyll *a* than in PBPs in Δ*ccaA*^KD^, while Δ*ccaA* + *cahB1* broadly recapitulates WT-like growth and pigmentation with statistically insignificant deviations. Finally, a strong increase in minimum cellular fluorescence F_o_ was observed in Δ*ccaA*^KD^ relative to WT (*p* = 1.6 × 10^−5^) which was fully recovered in Δ*ccaA* + *cahB1* complementation strains (Figure 2d). A significant reduction in maximum photosystem II quantum yield approximate QY_max_ observed in Δ*ccaA*^KD^ (*p* = 2.6 × 10^−8^) was partially recovered in Δ*ccaA* + *cahB1* (*p* = 2.6 × 10^−5^), while a slight yet statistically significant reduction in QY_max_ was also induced by expressing *cahB1* in the WT background (*p* = 4.7 × 10^−3^) (Figure 2d).

Taken together, these data suggest that the *cahB1* gene has a stimulating effect on photoautotrophic cell growth when added to WT cells and is capable of largely complementing the physiological defects caused by CcaA depletion. Thus, CahB1 is likely capable of functionally replacing carboxysomal β-CA in *Synechocystis*.

### 2.3. CahB1 Localizes to the Carboxysome in Synechocystis

**Figure 3:**
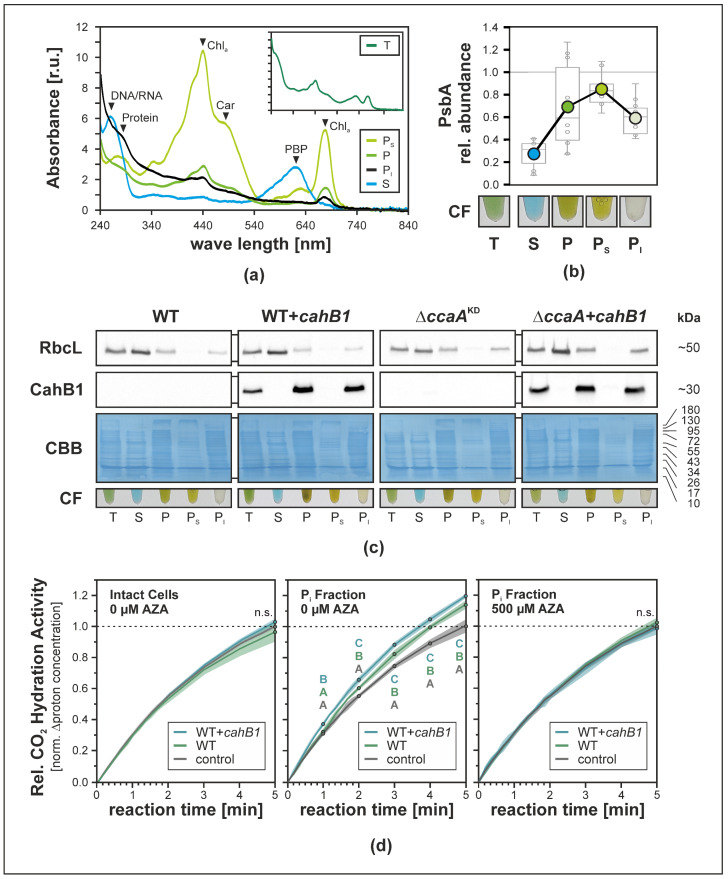
CahB1 localizes to a carboxysome-bearing and CA-active cell fraction in *Synechocystis*. (**a**) UV/Vis absorbance spectra of *Synechocystis* cell fractions. Total (T), soluble (S), pelletable (P), and α-/β-DDM solubilizable (P_S_) and insolubilizable (P_I_) subfractions of P. Traces represent average absorbance spectra adjusted to total protein content for all processed samples (*n* = 12; *n* = 3 for each genotype). Black arrowheads indicate compound correspondences of local absorbance maxima. [r.u.], relative units; Chl_a_, chlorophyll *a*; Car, carotenoids; PBP, phycobiliproteins. (**b**) Relative cell fraction (CF) contents of PsbA (D1) approximated by immunoblot ECL signal quantification. Coloured dots: average signal strength relative to T-fraction signals; boxplots: data points for *n* = 12 samples. Outliers beyond average ±1.5-fold standard deviation were excluded from the analysis. Representative samples of obtained CFs are shown. (**c**) Immunoblot analyses of RbcL and CahB1 accumulation in CFs. Sample input equilibrated based on Bradford assay protein content estimates; Coomassie Brilliant Blue (CBB) staining of PVDF membranes provided as loading control. (**d**) Relative CA activity of intact cells (left) and P_I_ cell fractions of WT and WT + *cahB1*. P_I_ CA activity was measured in absence (middle) and presence (right) of 500 µM acetazolamide (AZA). Averages (graphs) and standard deviations (shaded areas) of *n* = 3 biological replicates. Uppercase letters indicate statistically significant differences (*p* ≤ 0.05) in relative CO_2_ hydration rates according to multiple simultaneous comparisons in post hoc Bonferroni–Holm-corrected Tukey HSD tests after statistically significant among-group differences were detected by one-way ANOVA (**d** Intact Cells) *p* = 0.194; (**d** P_I_ 0 µM AZA) *p* = 0.005; (**d** P_I_ 500 µM AZA) *p* = 0.319; n.s., not significant.

In *Sodalinema gerasimenkoae*, CahB1 has been described to localize to the outer membrane/cell envelope fraction exclusively [26]. By virtue of complementing the Δ*ccaA* mutation in *Synechocystis*, however, localization of CahB1 to the carboxysome had to be assumed. To assess the subcellular localization of CahB1 in *Synechocystis*, cells were fractionated and CahB1 protein accumulation was traced by immunoblot assay using a specific peptide antibody raised against CahB1. Cells were broken mechanically using glass beads and a swing mill, and separated into total (T), soluble (S) and pelletable (P) fractions by centrifugation. Membrane proteins of the P fraction were then solubilized using a mixture of *n*-dodecyl-α/β-D-maltosides to obtain solubilizable (P_S_) and insolubilizable (P_I_) subfractions of the pelletable fraction. The obtained cell fractions displayed pronounced differences in their coloration and UV/Vis absorbance spectra (Figure 3a,b), as well as relative PsbA (i.e., D1) thylakoid marker protein content (i.e., relative depletion in fractions S and P_I_, and relative enrichment in fractions P and P_S_) (Figure 3b). The CahB1 protein could only be detected in cell fractions containing insolubilizable, pelletable components (i.e., fractions T, P, P_I_; Figure 3c). It was co-accumulated with a non-soluble and insolubilizable subpopulation of the RuBisCO large subunit protein RbcL (Figure 3c), suggesting co-localization of CahB1 with carboxysome-encapsulated cellular RuBisCO [33].

CO_2_ hydration assays commonly used to assess CA activity [6] showed CA-inhibitor-sensitive enhancements of CO_2_ hydration rates in P_I_ fractions of both WT and WT + *cahB1*, which was observed via proton release and a concordant drop in pH over a measuring period of five minutes (Figure 3d). CO_2_ hydration rates in WT + *cahB1* P_I_ fractions were found to be increased above WT levels and were significantly higher than both the no-sample control (*p* = 6.6 × 10^−3^) and WT sample rates (*p* = 1.3 × 10^−2^) starting after one minute, while the measured WT P_I_ fraction activity was only found to be significantly different from that of the no-sample control after two minutes (*p* = 2.2 × 10^−3^) and onward (Figure 3d middle). Meanwhile, for the entire measuring period of five minutes, no elevation above the uncatalyzed background rates was observed in intact cells assayed for extracellular CA activity (Figure 3d left) or acetazolamide-treated P_I_ fraction samples (Figure 3d right). These findings suggest that in *Synechocystis* CahB1 localizes to a cellular subfraction that harbours carboxysomes, and that the enzymatic activity of CahB1 is restricted to the said fraction rather than to the cell surface as would be expected based on previous reports from *Sodalinema gerasimenkoae* [24,25,26].

### 2.4. The Sodalinema gerasimenkoae Genome Lacks Genes for Carboxysomal CAs Other Than CahB1

**Figure 4:**
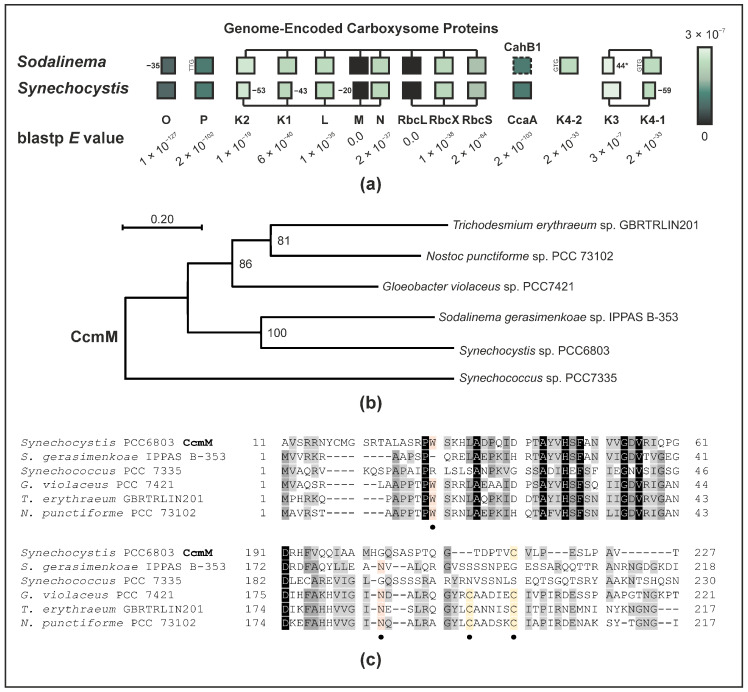
The *Sodalinema gerasimenkoae* genome encodes all essential carboxysomal proteins but lacks evidence of a functional carboxysomal carbonic anhydrase. (**a**) Relative distribution and operon structures of *Sodalinema gerasimenkoae* carboxysomal protein-coding genes. Protein extensions, truncations by premature stop codons (*), as well as usage of non-ATG start codons are indicated. Expectation (*E*) values of blastp searches against *Synechocystis* sp. PCC6803 homologues are listed and colour coded. (**b**) Maximum likelihood (log likelihood −8237.17) phylogenetic tree reconstruction of tentatively active and inactive CcmM γ-carbonic anhydrase proteins from different cyanobacteria as according to Peña and coworkers [8]. Numbers next to nodes indicate bootstrap support over 200 replicates. Branch lengths represent the number of substitutions per site (see scale bar). The analysis involved 6 amino acid sequences with a total of 899 positions in the final dataset. (**c**) Protein sequence alignment of two regions tentatively crucial to CcmM γ-carbonic anhydrase activity. Four presumably functionally important residues (marked by ●; [8]) are indicated: a structurally important tryptophane (W) and asparagine (N) residue are highlighted in orange; a pair of presumably catalytic cysteine (C) residues is highlighted in yellow. Conserved positions (black), highly similar positions (>80% similarity/identity; dark grey), and similar positions (>60% similarity/identity; light grey) are highlighted.

A genome sequence of the recently re-assigned *Sodalinema gerasimenkoae* IPPAS B-353 [30] has previously been published under the alias of *Microcoleus chthonoplastes* strain IPPAS B-353 (alias *Microcoleus chthonoplastes* strain IPPAS B-270) [31]. This genome sequence raises several additional questions as it encodes all structural subunits commonly forming a β-carboxysome [31,34], with very few atypical features to be observed in the predicted protein-coding gene sequences (Figure 4a). Unlike in *Synechocystis* where CcmK proteins are C-terminally truncated by 40–50 amino acid residues, the *Sodalinema gerasimenkoae* shell proteins CcmK1 and CcmK2 display typical lengths of 103 and 112 amino acid residues [35], respectively. Two near-identical copies of *ccmK4* are found within the *ccmK3/4* operon (designated *ccmK4-1*) and as a standalone satellite locus (designated *ccmK4-2*), which likewise display canonical lengths of 119 and 120 amino acid residues, respectively. The *ccmK3* gene meanwhile appears to be subject to a frameshift mutation resulting in a premature stop codon at position 44. As mentioned previously, the genome lacks any other *ccaA* homologue besides CahB1 that could serve as carboxysomal β-CA. While some beta-cyanobacteria are known not to possess *ccaA* homologues, in exchange their *ccmM* gene appears to encode an enzymatically active γ-CA [8]. CcmM of *Sodalinema gerasimenkoae* is highly homologous to *Synechocystis* CcmM and phylogenetically clusters with the latter rather than any other CcmM proteins, however (Figure 4b). Similar to in *Synechocystis*, CcmM in *Sodalinema gerasimenkoae* does not conserve several amino acid residues that are presumably essential to the CcmM’s γ-CA activity (Figure 4c); therefore, it is unlikely that it represents an active γ-CA enzyme.

In summary, these findings lead us to conclude that the *Sodalinema gerasimenkoae* β-carboxysome may function in an unusual way, presumably lacking an active CA enzyme, while the genome encodes a CcaA-homologous and β-carboxysome-compatible CA, CahB1. The mode of CahB1 secretion to the extracellular space in *Sodalinema gerasimenkoae* and the reasons for its apparent limitations in *Synechocystis* remain to be elucidated, just like the functional properties of the tentatively CA-less carboxysome in *Sodalinema gerasimenkoae*.

## 3. Discussion

The data presented in this study strongly suggest that *Sodalinema gerasimenkoae* CahB1 acts as a functional carboxysomal β-CA in *Synechocystis*, which can genetically and physiologically compensate for the loss of the endogenous *ccaA*/CcaA (Figure 1, Figure 2 and Figure 3). This is in line with the close sequence similarity that CahB1 bears to *Synechocystis* CcaA (Figure 1; [26]) and multiple reports of its functionality as an active and efficient CA enzyme [26,27,28]. Depletion of CcaA in the Δ*ccaA*^KD^ mutant results in a pronounced increase in minimal fluorescence, F_o_ (Figure 2d), which likely indicates a limitation of the acceptor side upon impaired carbon fixation [36,37,38], although the convolution of fluorescence from chlorophyll *a* with that from phycobiliprotein impair direct interpretation [39]. While Δ*ccaA*^KD^ shows overall reduced cellular pigment contents, cellular chlorophyll *a* levels appear to be disproportionately reduced compared to cellular phycobiliproteins (Figure 2b,c). This suggests the increase in F_o_ in Δ*ccaA*^KD^ may in part be attributed to a larger contribution of (allo-)phycocyanin fluorescence. Still, as both cellular chlorophyll *a* and phycobiliprotein levels are reduced, the observed increase in F_o_ is likely to underestimate the actual photosynthetic electron sink limitation caused by CcaA depletion. This limitation could be almost completely alleviated by the expression of CahB1, as indicated by the restored culture growth (Figure 2a), pigmentation (Figure 2b,c), and relative F_o_ (Figure 2d) in the complemented strains, which clearly indicates functional complementation of Δ*ccaA* by the introduced *cahB1* gene.

Our findings in the heterologous *Synechocystis* test system contrast sharply with the proposed location of CahB1 outside of the *Sodalinema gerasimenkoae* carboxysome and outside the cytosol altogether [25,26]. While no extracellular CA activity could be observed upon expression of CahB1 in *Synechocystis*, a slight yet statistically significant enhancement of CA activity in the carboxysome-enriched cell fraction P_I_ of WT + *cahB1* cells as compared to the parental WT cells (Figure 3d) may account for the enhanced growth observed in the said expression strains (Figure 2b). As no enhanced growth beyond WT levels was observed in Δ*ccaA + cahB1* complementation strains, growth enhancement is unlikely to stem from the presence of CahB1 alone and may either result from synergy effects in a cellular hybrid population of the two carboxysomal β-CAs, or from an overall increased level of cellular carboxysomal β-CA. In CahB1-expressing strains, the slight reduction in photosystem II maximum quantum efficiency estimate QYmax we observed (Figure 2d) may simply be attributed to the use of chloramphenicol as a positive selection marker. Chloramphenicol is a potent protein biosynthesis inhibitor commonly used to suppress the reparation of photodamaged D1 protein [40] and may therefore affect QYmax even in the presence of chloramphenicol acetyl transferase enzyme. Given the enhanced growth phenotype of WT + *cahB1* strains, however, we consider this reduction in QYmax to be largely inconsequential. Future experiments will be required to dissect the underlying mechanism of enhanced growth in *cahB1* and whether this feature is applicable to other model systems and cyanobacterial production strains.

Isolation of intact carboxysomes was attempted using a gentle approach employing glass beads and relatively weak detergents instead of the conventional French-press/Triton-Percoll methods [41], as the latter have been found to disrupt the carboxysome shell by removing large proportions of the CcmK2 shell component [42], thus potentially releasing any carboxysome-encapsulated CahB1 if it associates with the carboxysomal shell similar to CcaA [7]. Successful enrichment of carboxysomes in the P_I_ fraction is indicated by the complete transfer of RbcL from the P fraction into the P_I_ fraction (Figure 3c), with a large subpopulation of cytosolic free-standing RuBisCO complexes and assembly intermediates, as indicated by the RbcL signal in the S fraction being well in line with the previous reports [33]. The low catalytic activity observed in CO_2_ hydration assays (Figure 3d) is similar to what has been observed in intact carboxysomes, whose CA catalytic activity is much lower than that of ruptured carboxysomes [43] or purified CcaA [7]. This is likely due to the intact microcompartment shell acting as a strong diffusive barrier which, under physiological conditions, is hypothesized to prevent leakage of internally generated CO_2_ from carboxysomes while simultaneously protecting encapsulated RuBisCO from cytosolic O_2_ [44]. The complete inhibition of P_I_-fraction CA activity by acetazolamide (AZA) is in line with previous reports of CahB1 being susceptible to AZA [27]. To our knowledge, susceptibility of *Synechocystis* CcaA to inhibitors has not been investigated in detail so far but recombinant CcaA is enzymatically active [7,11], and AZA-tolerant *Synechocystis* mutants have been described and hypothesized to harbour mutations in CA [45], even prior to the initial cloning of the *ccaA* gene [10]. Moreover, the high level of homology between CcaA and CahB1 (Figure 1; [26]), and the fact that CcaA is the only essential CA identified in *Synechocystis* so far [16,23], strongly suggest that CcaA would also be susceptible to AZA inhibition. Thus, the P_I_ fraction is considered to likely contain enriched and intact carboxysomes with whom active CahB1 enzyme co-precipitates in both WT *+ cahB1* and Δ*ccaA + cahB1* (Figure 3c). We hence conclude that heterologously-expressed CahB1 is likely to play the structural and physiological roles of the *Synechocystis* β-carboxysome, which is further supported by our observation of the *cahB1* gene allowing for complete segregation of Δ*ccaA*^KD^ mutants (Figure 1c).

The *Sodalinema gerasimenkoae* genome appears to encode a full set of functional β-carboxysomal genes [30,31]. While the *ccmK3* gene is likely functionally disrupted, the shell protein CcmK3 has been shown to be dispensable, and knockout mutations do not severely affect the growth in *Synechococcus* sp. PCC7942 [46,47]. Still, its absence may result in assembled carboxysome shells with altered properties, e.g., considering the pH-dependent metabolite permeability of the carboxysome shell [47]. Much more intriguingly, the *Sodalinema gerasimenkoae* genome lacks potential gene(s) encoding carboxysomal CA besides *cahB1*, (Figure 4; [30,31]). In both α- and β-type carboxysomes, a functional CA enzyme is widely considered functionally integral [48,49], although *Synechocystis* carboxysomes have been observed to structurally assemble even in the absence of CcaA [16]. This raises the question of how the *Sodalinema gerasimenkoae* carboxysome functions and whether there is an unknown component that can substitute the β-CA CahB1 and/or the γ-CA CcmM [8]. Thus, the *Sodalinema gerasimenkoae* carboxysome appears highly atypical in this regard and may invite various lines of future functional investigation.

Lastly, the mode of secretion employed to target CahB1 to the outer membrane in *Sodalinema gerasimenkoae* remains elusive. *Synechocystis* sp. PCC6803 seemingly sequesters active CahB1 to the carboxysome, yet comparative analyses of the N-terminal region of CahB1/CcaA may hint at an underlying mechanism. The CahB1 N-terminus, like all other investigated homologues except for *Synechocystis* CcaA, starts with an MKK tripeptide. Unlike its homologues, the first 20 amino acid residues do not contain any negatively charged residues, however (Figure 1b), making it more similar to the positively charged *n*-region and the non-polar *h*-region of other cyanobacterial CA secretion peptides, such as that of *Synechococcus* EcaA [19]. While an MKK-tripeptide close to the N-terminus has been described to facilitate protein secretion in a *Bacillus*-derived synthetic secretion signal [50], negatively charged amino-acid residues are commonly absent from pre-cleavage site secretion signal sequences in eukaryotes, Gram-negative, and Gram-positive bacteria [51]. Thus, the CahB1 N-terminus may serve as a kind of proto-secretion signal that is sufficient to elicit protein secretion in the original *Sodalinema gerasimenkoae* system, but not in *Synechocystis*. CahB1 may thus serve as an interesting case study on how secretion signals can evolve out of previously non-secreted N-terminal sequences. On the other hand, as CcaA is hypothesized to be a later acquisition by horizontal gene transfer [8], the originally acquired beta-CA may have indeed been a secreted enzyme that evolved not to be secreted but rather to be sequestered to the carboxysome in β-cyanobacteria. In this case, CahB1 may represent a relic CcaA isoform whose secretion has not (yet) been evolutionarily suppressed in *Sodalinema gerasimenkoae*.

## 4. Materials and Methods

**Taxonomy**

The organism from which the *cahB1* gene was originally isolated has undergone multiple taxonomic re-assignments. Originally being referred to as *Microcoleus chthonoplastes* IPPAS B-270 [25,26], synonymous to *Microcoleus chthonoplastes* IPPAS B-353 [31], the species was re-assigned to the genus *Coleofasciculus* [52] and then re-named *Sodalinema gerasimenkoae* IPPAS B-353 [21,30]. For the scope of this study, we refer to it as per the latest designation, i.e., *Sodalinema gerasimenkoae* IPPAS B-353.

**Sequence Analysis, Alignment, and Phylogenetic Reconstruction**

Protein sequences were obtained from NCBI GenBank [53] using Blastp (https://blast.ncbi.nlm.nih.gov/Blast.cgi, accessed on 29 November 2022). Multiple protein sequence alignments were routinely generated using MUSCLE [54] with default parameters as implemented in MEGA X [55]. Maximum-likelihood (ML) phylogenetic reconstructions were conducted upon determining the respective best-fit amino-acid substitution model [56] using the model test tool implemented in MEGA X. *Sodalinema gerasimenkoae* genome annotation entries [30,31] were browsed and analysed using Geneious Prime 2022.0.1 (https://www.geneious.com, accessed on 22 February 2022). Signal peptide prediction was performed using SignalP v. 6.0 [57] and DeepTMHMM [58]. For signal peptide prediction outputs, see Supplementary Material File S1.

***Synechocystis* cultivation and mutant generation**

Cyanobacterial cells were routinely grown on BG11 media [59]. Stock cultures were grown on BG11 agar (0.8% *w/v*) plates containing 10 mM TES-KOH and 4 g L^−1^ sodium thiosulfate. Assay cultures were grown for seven days in liquid BG11 media at 25 °C under continuous illumination of 50 µmol photons m^−2^ s^−1^ white fluorescent light, continuous orbital agitation (65 rpm), and under exclusively atmospheric CO_2_ supply. Glucose-tolerant non-motile *Synechocystis* sp. PCC6803 wildtype cells were obtained from the Leister lab (LMU Munich). Mutant strains were generated by natural transformation and homologous recombination as described previously [60].

*Sodalinema gerasimenkoae cahB1* was cloned from a genomic library fragment provided by Elena Kupriyanova [26]. *Synechocystis* genes, promoters, and sequences for homologous recombination were amplified from genomic DNA extracts using HS VeriFi^TM^ high-fidelity polymerase (PCR Biosystems Ltd., London, UK). Constructs for expression of CahB1 (pNS_cahB1_CmR) and KO of *Synechocystis ccaA* (pΔccaA-KanR) were cloned by Gibson assembly [61] (New England Biolabs Inc., Ipswich, MA, USA). For DNA vector sequences, see Supplementary Material File S2.

Transformants were selected and segregated on incrementally increasing antibiotic concentrations in solid media, reaching final working concentrations of 100 µg mL^−1^ for kanamycin, and 15 µg mL^−1^ for chloramphenicol. Genotyping PCR was performed using Phire Plant Direct PCR Master Mix (ThermoFisher Scientific Inc., Waltham, MA, USA).

***Synechocystis* photosynthetic measurements**

Minimal fluorescence F_o_ and photosystem II maximum quantum yield QYmax were measured using a FluorCam 800 MF (Photon Systems Instruments, Drásov, Czech Republic) as previously described [62]. Culture samples grown as outlined above were harvested, washed with BG11 media, and adjusted to a final OD_730nm_ = 75. Aliquots of 10 µL were then dropped onto BG11 agar plates and incubated under previously applied growth conditions for three hours prior to measurement.

***Synechocystis* pigment extraction and quantification**

Hydrophobic pigments were extracted and quantified as previously described [62,63].

Phycobiliprotein contents were estimated based on whole-cell UV/Vis absorbance spectra obtained at seven days past inoculation using a NanoDrop™ 2000c spectrophotometer (ThermoFisher Scientific Inc., Waltham, MA, USA) as previously described [64,65]. In preparation, whole-cell spectra were baseline corrected to absorbance values of 0.00 at λ = 750 nm (A_750_) with A_550_ being set to 0.2 × A_644_ in accordance with established *Synechocystis* phycobilisome absorbance spectra [66] (see Supplementary Materials File S3).

***Synechocystis* cell fractionation**

*Synechocystis* cells were harvested 7 days past inoculation by centrifugation, broken, and separated into total (T), soluble (S), and pelletable (P) fractions by centrifugation (4 °C, 16,000 rcf; 30 min) as described previously [67]. Membrane proteins of the P fraction were solubilized on ice for 10 min under occasional mixing using a mixture of *n*-dodecyl-α/β-D-maltosides (α: 1.4% *w/v*; β: 1.0% *w/v* final concentrations; Anatrace Products LLC, Maumee, OH, USA) to obtain solubilizable (P_S_) and insolubilizable (P_I_) subfractions of the pelletable fraction, which were subsequently separated by centrifugation (4 C; 16,000 rcf; 30 min). P_I_ fraction pellets were then washed and re-pelleted (4 °C; 16,000 rcf; 15 min) twice with 500 µL tricine buffer [67] containing 1.4% (*w/v*) and 1.0% (*w/v*) *n*-dodecyl-α/β-D-maltosides, respectively, and finally resuspended in 50 µL of tricine buffer. UV/Vis absorbance spectra of obtained cell fractions were measured using a NanoDrop™ 2000c spectrophotometer (ThermoFisher Scientific Inc., Waltham, MA, USA), and protein contents were approximated by Bradford protein assay (Nacalai Tesque Inc., Kyoto, Japan) prior to performing SDS-PAGE/immunoblot and CO_2_ hydration assays.

**Immunoblot analyses**

A specific peptide antibody against *Sodalinema gerasimenkoae* CahB1 (aa185-198:C-DVEELVPGHRQSSA) was raised in rabbit and affinity-purified by BioGenes GmbH (Berlin, Germany). Global antibodies against RbcL (AS03 037) and PsbA (C-terminus; AS05 084A) were obtained from Agrisera AB (Vännäs, Sweden). Size separation of protein samples by Tris/Tricine SDS PAGE and subsequent immunoblot analyses/imaging by ECL were performed as described previously [62]. PpsbA ECL signal quantification was performed using ImageJ [68].

**CO_2_ hydration assay**

Carbonic anhydrase activity was assessed via observation of CO_2_-hydration-driven pH drop in 25 mM K_2_HPO_4_ (pH 8.45) assay buffer as previously described [6]. For intact cell extracellular CA activity measurements, OD_730nm_ = 5.0 cell equivalents were harvested, washed, and resuspended in assay buffer prior to measurement. For P_I_ fraction activity measurements, OD_730nm_ = 10.0 cell equivalents were fractionated as described above, and P_I_ fractions were equilibrated for equal total protein content as approximated by Bradford protein assay (Nacalai Tesque Inc., Kyoto, Japan) with the lowest concentration sample being adjusted to 100 µL. An amount of 50 µL of P_I_ fraction was then assayed for CA activity in absence and presence of 500 µM acetazolamide (Sigma-Aldrich, St. Louis, MO, USA). Assays were conducted on ice at 4 °C for 5 min, with a total assay buffer plus sample volume of 14.5 mL and 500 µL of ice-cold CO_2_-saturated H_2_O. Measurements were taken in 10 s intervals for the first 2 min, and in 60 s intervals for another three minutes.

**Statistical Analyses**

Charts were created using Microsoft Office Excel 365. For boxplots, internal datapoints are represented as dots. Horizontal lines represent the median, crosses represent average values, and boxes indicate the 25th and 75th percentiles. Whiskers extend 1.5-fold the interquartile range and outliers. Statistically significant among-group differences were tested for by one-way ANOVA, followed by post hoc Tukey HSD (honest significant differences) tests with Bonferroni–Holm *p*-value correction for multiple comparison. Significant differences according to multiple simultaneous post hoc comparisons are routinely indicated by uppercase/lowercase letters denoting the resultant groups of not significantly different (same letter) and significantly different (different letter) samples. Analyses were performed using the one-way ANOVA with post hoc test tool as implemented by Navendu Vasavada (https://astatsa.com/, accessed on 9 December 2022). All statistical analysis results are detailed in the corresponding source data sheets (see Supplementary Materials File S3).

## 5. Conclusions

The physiological role of extracellular carbonic anhydrases in cyanobacteria remains rather elusive. *Sodalinema gerasimenkoae* CahB1 represents an intriguing case study, as its activity may be crucial to efficient carbon uptake within alkaliphilic biofilms, but its mode of secretion and any functional compensation for its absence from the carboxysome are hard to reconcile given its genomic context and its demonstrated functionality as a carboxysomal β-CA in *Synechocystis*. Its close homology to and functional interchangeability with *Synechocystis* CcaA may hint at a relatively recent and subtle evolutionary divergence that allows for its re-localization from the carboxysome to the outer cell membrane. The exact mechanism underlying this evolutionary shift and its correlation with respect to the carboxysome function remain to be deciphered. This study provides the first stepping stone to elucidating the origin of this highly atypical and—to our knowledge—singular functional extracellular β-CA in cyanobacteria.

## Data Availability

All experimental data reported in this study are made available in the Supplementary Materials File S3 accompanying this report. For any further information, please refer to the corresponding author.

## Supplementary Materials

The following are available online at: https://www.mdpi.com/article/10.3390/plants12020265/s1, File S1: CcaA-CahB1-EcaB secretion-signal-prediction software outputs of SignalP v. 6.0 and DeepTMHMM (File S1_MDPI PLANTS CahB1_Cyanobacterial beta-CA Secretion Signal Predictions); File S2: pΔccaA-KanR and pNS_cahB1-CmR vector maps (File S2_MDPI PLANTS CahB1_vector sequences); File S3: Microsoft Excel workbook including inventory, DNA primer sequences, raw data and statistical analyses of data presented in Figure 1, Figure 2 and Figure 3 (File S3_MDPI PLANTS CahB1_raw data and analyses); File S4: Unedited images of DNA Gels (Figure 1c) and protein blots (Figure 3b,c) (File S4_MDPI PLANTS CahB1_Original Images DNA Gels and Protein Blots).

## References

[1] Hirakawa Y., Senda M., Fukuda K., Yu H.Y., Ishida M., Taira M., Kinbara K., Senda T. (2021). Characterization of a novel type of carbonic anhydrase that acts without metal cofactors. BMC Biol..

[2] Badger M.R., Price G.D., Long B.M., Woodger F.J. (2006). The environmental plasticity and ecological genomics of the cyanobacterial CO_2_ concentrating mechanism. J. Exp. Bot..

[3] Price G.D., Howitt S.M. (2011). The cyanobacterial bicarbonate transporter BicA: Its physiological role and the implications of structural similarities with human SLC26 transporters. Biochem. Cell Biol..

[4] Kupriyanova E., Pronina N., Los D. (2017). Carbonic anhydrase—A universal enzyme of the carbon-based life. Photosynthetica.

[5] Wang Y., Stessman D.J., Spalding M.H. (2015). The CO_2_ concentrating mechanism and photosynthetic carbon assimilation in limiting CO 2: How Chlamydomonas works against the gradient. Plant J..

[6] Miller A.G., Colman B. (1980). Evidence for HCO_3_ Transport by the Blue-Green Alga (Cyanobacterium) Coccochloris peniocystis. Plant Physiol..

[7] Cot S.S., So A.K., Espie G.S. (2008). A multiprotein bicarbonate dehydration complex essential to carboxysome function in cyanobacteria. J. Bacteriol..

[8] Pena K.L., Castel S.E., de Araujo C., Espie G.S., Kimber M.S. (2010). Structural basis of the oxidative activation of the carboxysomal gamma-carbonic anhydrase, CcmM. Proc. Natl. Acad. Sci. USA.

[9] McGurn L.D., Moazami-Goudarzi M., White S.A., Suwal T., Brar B., Tang J.Q., Espie G.S., Kimber M.S. (2016). The structure, kinetics and interactions of the β-carboxysomal β-carbonic anhydrase, CcaA. Biochem. J..

[10] So A.K., Espie G.S. (1998). Cloning, characterization and expression of carbonic anhydrase from the cyanobacterium Synechocystis PCC6803. Plant Mol. Biol..

[11] So A.K.C., Van Spall H.G.C., Coleman J.R., Espie G.S. (1998). Catalytic exchange of ^18^O from ^13^C^18^O-labelled CO_2_ by wild-type cells and ecaA, ecaB, and ccaA mutants of the cyanobacteria Synechococcus PCC7942 and Synechocystis PCC6803. Can. J. Bot..

[12] McGinn P.J., Price G.D., Maleszka R., Badger M.R. (2003). Inorganic carbon limitation and light control the expression of transcripts related to the CO_2_-concentrating mechanism in the cyanobacterium *Synechocystis* sp. strain PCC6803. Plant Physiol..

[13] Wang H.-L., Postier B.L., Burnap R.L. (2004). Alterations in global patterns of gene expression in *Synechocystis* sp. PCC 6803 in response to inorganic carbon limitation and the inactivation of ndhR, a LysR family regulator. J. Biol. Chem..

[14] Eisenhut M., Von Wobeser E.A., Jonas L., Schubert H., Ibelings B.W., Bauwe H., Matthijs H.C., Hagemann M. (2007). Long-term response toward inorganic carbon limitation in wild type and glycolate turnover mutants of the cyanobacterium *Synechocystis* sp. strain PCC 6803. Plant Physiol..

[15] Price G.D., Badger M.R., Woodger F.J., Long B.M. (2008). Advances in understanding the cyanobacterial CO_2_-concentrating-mechanism (CCM): Functional components, Ci transporters, diversity, genetic regulation and prospects for engineering into plants. J. Exp. Bot..

[16] So A.K., John-McKay M., Espie G.S. (2002). Characterization of a mutant lacking carboxysomal carbonic anhydrase from the cyanobacterium Synechocystis PCC6803. Planta.

[17] Li T., Sharp C.E., Ataeian M., Strous M., de Beer D. (2018). Role of Extracellular Carbonic Anhydrase in Dissolved Inorganic Carbon Uptake in Alkaliphilic Phototrophic Biofilm. Front. Microbiol..

[18] Soltes-Rak E., Mulligan M.E., Coleman J.R. (1997). Identification and characterization of a gene encoding a vertebrate-type carbonic anhydrase in cyanobacteria. J. Bacteriol..

[19] Kupriyanova E.V., Sinetova M.A., Bedbenov V.S., Pronina N.A., Los D.A. (2018). Putative extracellular alpha-class carbonic anhydrase, EcaA, of Synechococcus elongatus PCC 7942 is an active enzyme: A sequel to an old story. Microbiology.

[20] Kupriyanova E.V., Sinetova M.A., Mironov K.S., Novikova G.V., Dykman L.A., Rodionova M.V., Gabrielyan D.A., Los D.A. (2019). Highly active extracellular alpha-class carbonic anhydrase of *Cyanothece* sp. ATCC 51142. Biochimie.

[21] Kupriyanova E.V., Sinetova M.A., Leusenko A.V., Voronkov A.S., Los D.A. (2022). A leader peptide of the extracellular cyanobacterial carbonic anhydrase ensures the efficient secretion of recombinant proteins in Escherichia coli. J. Biotechnol..

[22] Fulda S., Huang F., Nilsson F., Hagemann M., Norling B. (2000). Proteomics of *Synechocystis* sp. strain PCC 6803. Identification of periplasmic proteins in cells grown at low and high salt concentrations. Eur. J. Biochem..

[23] Sun N., Han X., Xu M., Kaplan A., Espie G.S., Mi H. (2019). A thylakoid-located carbonic anhydrase regulates CO_2_ uptake in the cyanobacterium *Synechocystis* sp. PCC 6803. New Phytol..

[24] Kupriyanova E.V., Lebedeva N.V., Dudoladova M.V., Gerasimenko L.M., Alekseeva S.G., Pronina N.A., Zavarzin G.A. (2003). Carbonic Anhydrase Activity of Alkalophilic Cyanobacteria from Soda Lakes. Russ. J. Plant Physiol..

[25] Kupriyanova E.V., Markelova A.G., Lebedeva N.V., Gerasimenko L.M., Zavarzin G.A., Pronina N.A. (2004). Carbonic Anhydrase of the Alkaliphilic Cyanobacterium Microcoleus chthonoplastes. Microbiology.

[26] Kupriyanova E.V., Sinetova M.A., Markelova A.G., Allakhverdiev S.I., Los D.A., Pronina N.A. (2011). Extracellular beta-class carbonic anhydrase of the alkaliphilic cyanobacterium Microcoleus chthonoplastes. J. Photochem. Photobiol. B.

[27] Vullo D., Kupriyanova E.V., Scozzafava A., Capasso C., Supuran C.T. (2014). Anion inhibition study of the β-carbonic anhydrase (CahB1) from the cyanobacterium Coleofasciculus chthonoplastes (ex-Microcoleus chthonoplastes). Bioorganic Med. Chem..

[28] Heuer J., Kraus Y., Vucak M., Zeng A.P. (2022). Enhanced sequestration of carbon dioxide into calcium carbonate using pressure and a carbonic anhydrase from alkaliphilic Coleofasciculus chthonoplastes. Eng. Life Sci..

[29] Hewett-Emmett D., Tashian R.E. (1996). Functional diversity, conservation, and convergence in the evolution of the alpha-, beta-, and gamma-carbonic anhydrase gene families. Mol. Phylogenet Evol..

[30] Samylina O.S., Sinetova M.A., Kupriyanova E.V., Starikov A.Y., Sukhacheva M.V., Dziuba M.V., Tourova T.P. (2021). Ecology and biogeography of the ‘marine Geitlerinema’ cluster and a description of *Sodalinema orleanskyi* sp. nov., *Sodalinema gerasimenkoae* sp. nov., *Sodalinema stali* sp. nov. and *Baaleninema simplex* gen. et sp. nov. (Oscillatoriales, Cyanobacteria). FEMS Microbiol. Ecol..

[31] Kupriyanova E.V., Cho S.M., Park Y.-I., Pronina N.A., Los D.A. (2016). The complete genome of a cyanobacterium from a soda lake reveals the presence of the components of CO_2_-concentrating mechanism. Photosynth. Res..

[32] Fukuzawa H., Suzuki E., Komukai Y., Miyachi S. (1992). A gene homologous to chloroplast carbonic anhydrase (icfA) is essential to photosynthetic carbon dioxide fixation by Synechococcus PCC7942. Proc. Natl. Acad. Sci. USA.

[33] Dai W., Chen M., Myers C., Ludtke S.J., Pettitt B.M., King J.A., Schmid M.F., Chiu W. (2018). Visualizing Individual RuBisCO and Its Assembly into Carboxysomes in Marine Cyanobacteria by Cryo-Electron Tomography. J. Mol. Biol..

[34] Kerfeld C.A., Melnicki M.R. (2016). Assembly, function and evolution of cyanobacterial carboxysomes. Curr. Opin. Plant Biol..

[35] Sommer M., Cai F., Melnicki M., Kerfeld C.A. (2017). beta-Carboxysome bioinformatics: Identification and evolution of new bacterial microcompartment protein gene classes and core locus constraints. J. Exp. Bot..

[36] Kautsky H., Appel W., Amann H. (1960). Chlorophyll fluorescence and carbon assimilation. Part XIII. The fluorescence and the photochemistry of plants. Biochem. Z.

[37] Maxwell K., Johnson G.N. (2000). Chlorophyll fluorescence—A practical guide. J. Exp. Bot..

[38] Kalaji H.M., Schansker G., Brestic M., Bussotti F., Calatayud A., Ferroni L., Goltsev V., Guidi L., Jajoo A., Li P. (2017). Frequently asked questions about chlorophyll fluorescence, the sequel. Photosynth. Res..

[39] Campbell D., Hurry V., Clarke A.K., Gustafsson P., Oquist G. (1998). Chlorophyll fluorescence analysis of cyanobacterial photosynthesis and acclimation. Microbiol. Mol. Biol. Rev..

[40] Treves H., Raanan H., Kedem I., Murik O., Keren N., Zer H., Berkowicz S.M., Giordano M., Norici A., Shotland Y. (2016). The mechanisms whereby the green alga Chlorella ohadii, isolated from desert soil crust, exhibits unparalleled photodamage resistance. New Phytol..

[41] Long B.M., Badger M.R., Whitney S.M., Price G.D. (2007). Analysis of carboxysomes from Synechococcus PCC7942 reveals multiple Rubisco complexes with carboxysomal proteins CcmM and CcaA. J. Biol. Chem..

[42] Rae B.D., Long B.M., Badger M.R., Price G.D. (2013). Functions, compositions, and evolution of the two types of carboxysomes: Polyhedral microcompartments that facilitate CO_2_ fixation in cyanobacteria and some proteobacteria. Microbiol. Mol. Biol. Rev..

[43] Heinhorst S., Williams E.B., Cai F., Murin C.D., Shively J.M., Cannon G.C. (2006). Characterization of the carboxysomal carbonic anhydrase CsoSCA from Halothiobacillus neapolitanus. J. Bacteriol..

[44] Turmo A., Gonzalez-Esquer C.R., Kerfeld C.A. (2017). Carboxysomes: Metabolic modules for CO_2_ fixation. FEMS Microbiol. Lett..

[45] Bédu S., Peltier G., Sarrey F., Joset F. (1990). Properties of a Mutant from Synechocystis PCC6803 Resistant to Acetazolamide, an Inhibitor of Carbonic Anhydrase. Plant Physiol..

[46] Rae B.D., Long B.M., Badger M.R., Price G.D. (2012). Structural determinants of the outer shell of beta-carboxysomes in Synechococcus elongatus PCC 7942: Roles for CcmK2, K3-K4, CcmO, and CcmL. PLoS ONE.

[47] Sommer M., Sutter M., Gupta S., Kirst H., Turmo A., Lechno-Yossef S., Burton R.L., Saechao C., Sloan N.B., Cheng X. (2019). Heterohexamers Formed by CcmK3 and CcmK4 Increase the Complexity of Beta Carboxysome Shells. Plant Physiol..

[48] Kimber M.S. (2014). Carboxysomal carbonic anhydrases. Subcell Biochem..

[49] Gee C.W., Niyogi K.K. (2017). The carbonic anhydrase CAH1 is an essential component of the carbon-concentrating mechanism in Nannochloropsis oceanica. Proc. Natl. Acad. Sci. USA.

[50] Low K.O., Jonet M.A., Ismail N.F., Illias R.M. (2012). Optimization of a Bacillus sp signal peptide for improved recombinant protein secretion and cell viability in Escherichia coli: Is there an optimal signal peptide design?. Bioengineered.

[51] Choo K.H., Ranganathan S. (2008). Flanking signal and mature peptide residues influence signal peptide cleavage. BMC Bioinform..

[52] Siegesmund M.A., Johansen J.R., Karsten U., Friedl T. (2008). Coleofasciculus gen. nov.(Cyanobacteria): Morphological and Molecular Criteria for Revision of the Genus microcoleus gomont 1. J. Phycol..

[53] Clark K., Karsch-Mizrachi I., Lipman D.J., Ostell J., Sayers E.W. (2016). GenBank. Nucleic Acids Res..

[54] Edgar R.C. (2004). MUSCLE: Multiple sequence alignment with high accuracy and high throughput. Nucleic Acids Res..

[55] Kumar S., Stecher G., Li M., Knyaz C., Tamura K. (2018). MEGA X: Molecular Evolutionary Genetics Analysis across Computing Platforms. Mol. Biol. Evol..

[56] Nei M., Kumar S. (2000). Molecular Evolution and Phylogenetics.

[57] Teufel F., Almagro Armenteros J.J., Johansen A.R., Gíslason M.H., Pihl S.I., Tsirigos K.D., Winther O., Brunak S., von Heijne G., Nielsen H. (2022). SignalP 6.0 predicts all five types of signal peptides using protein language models. Nat. Biotechnol..

[58] Hallgren J., Tsirigos K.D., Pedersen M.D., Armenteros J.J.A., Marcatili P., Nielsen H., Krogh A., Winther O. (2022). DeepTMHMM predicts alpha and beta transmembrane proteins using deep neural networks. bioRxiv.

[59] Waterbury J.B., Stanier R.Y. (1981). Isolation and growth of cyanobacteria from marine and hypersaline environments. The Prokaryotes.

[60] Viola S., Ruhle T., Leister D. (2014). A single vector-based strategy for marker-less gene replacement in *Synechocystis* sp. PCC 6803. Microb. Cell Fact..

[61] Gibson D.G., Young L., Chuang R.-Y., Venter J.C., Hutchison C.A., Smith H.O. (2009). Enzymatic assembly of DNA molecules up to several hundred kilobases. Nat. Methods.

[62] Dann M., Ortiz E.M., Thomas M., Guljamow A., Lehmann M., Schaefer H., Leister D. (2021). Enhancing photosynthesis at high light levels by adaptive laboratory evolution. Nat. Plants.

[63] Zavřel T., Sinetova M.A., Červený J. (2015). Measurement of Chlorophyll a and Carotenoids Concentration in Cyanobacteria. Bio-protocol.

[64] Rakhimberdieva M.G., Boichenko V.A., Karapetyan N.V., Stadnichuk I.N. (2001). Interaction of phycobilisomes with photosystem II dimers and photosystem I monomers and trimers in the cyanobacterium Spirulina platensis. Biochemistry.

[65] Luimstra V.M., Schuurmans J.M., de Carvalho C.F.M., Matthijs H.C.P., Hellingwerf K.J., Huisman J. (2019). Exploring the low photosynthetic efficiency of cyanobacteria in blue light using a mutant lacking phycobilisomes. Photosynth. Res..

[66] Zlenko D.V., Elanskaya I.V., Lukashev E.P., Bolychevtseva Y.V., Suzina N.E., Pojidaeva E.S., Kononova I.A., Loktyushkin A.V., Stadnichuk I.N. (2019). Role of the PB-loop in ApcE and phycobilisome core function in cyanobacterium *Synechocystis* sp. PCC 6803. Biochim. Biophys. Acta (BBA)-Bioenerg..

[67] Gandini C., Schmidt S.B., Husted S., Schneider A., Leister D. (2017). The transporter Syn PAM 71 is located in the plasma membrane and thylakoids, and mediates manganese tolerance in Synechocystis PCC 6803. New Phytol..

[68] Schneider C.A., Rasband W.S., Eliceiri K.W. (2012). NIH Image to ImageJ: 25 years of image analysis. Nat. Methods.

