# Supplementary material for: Extracellular CahB1 from Sodalinema gerasimenkoae IPPAS B-353 Acts as a Functional Carboxysomal β-Carbonic Anhydrase in Synechocystis sp. PCC6803
Source: Plants (Basel). 2023 Jan 6;12(2):265. doi: 10.3390/plants12020265 (PMC9865033; doi:10.3390/plants12020265)
Supplement: Supplementary file 1 [file plants-12-00265-s001.zip › File S1 _ MDPI PLANTS CahB1 _ Cyanobacterial beta-CA Secretion Signal Predictions REV.pdf]

Supplementary Materials File S1

CcaA/CahB1/EcaB N-terminal Secretion Signal Prediction Software Outputs

Concerning Figure 1 b:

Top: Protein sequence as extracted from NCBI Genbank
Middle: SignalP v. 6.0 graphic output (https://services.healthtech.dtu.dk/service.php?SignalP)
Bottom: DeepTMHMM graphic output (https://dtu.biolib.com/DeepTMHMM)

Secretion signal predictions CcaA/CahB1 and EcaB homologues

>WP_011055160.1 carbonic anhydrase [Synechococcus elongatus PCC7942]

MTVLTEVLEANQAYAAAFGEKGSLALPPARQFAILTCMDARLDPAQYAGLKEGDAHVIRNAGGRASDDAIRSLVISYKLLGTKEWFVIHHTDCGMEFFTNSVIGSLLANSLETAVLRSDGFTDIGEGPGSEEGKYIHWLTIENQTQSVVEDVQRIRRHPLVPSNIPIYGYLYDVRSGRLIEVPEATTAGVAR

Sequence
Prediction: Other

Protein type	Other	Signal Peptide (Sec/SPI)	Lipoprotein signal peptide (Sec/SPII)	TAT signal peptide (Tat/SPI)	TAT Lipoprotein signal peptide (Tat/SPII)	Pilin-like signal peptide (Sec/SPIII)
**Likelihood**	1	0	0	0	0	0

Download: PNG / EPS / Tabular

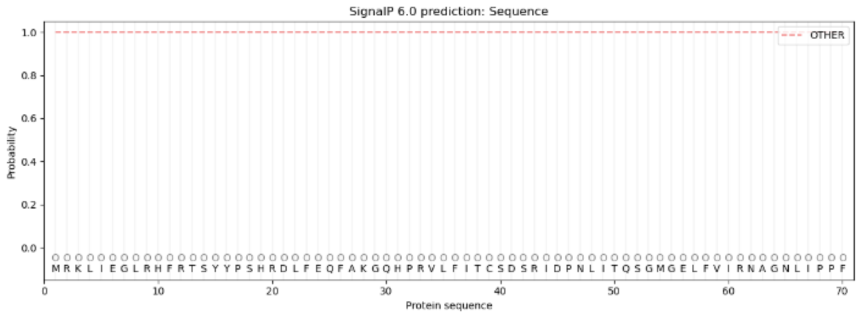

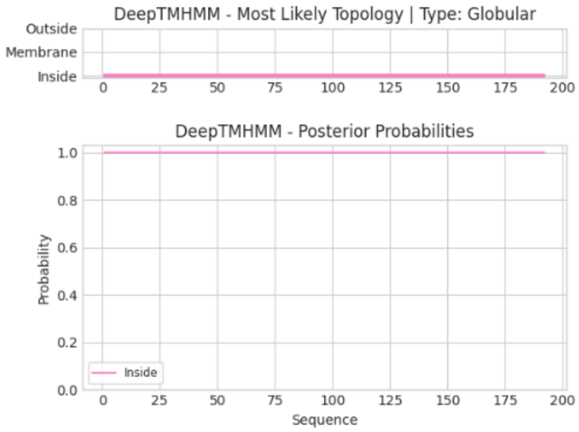


```
MTVLTEVLEANQAYAAAFGEKGSLALPPARQFAILTCMDARLDPAQYAGLKEGDAHVIRNAGGRASDDAIRSLVISYKLLGTKEWFVIHHTDCGMEFFTNSVIGSLLANSLETAVLRSDGFTDI
GEGPGSEEGKYIHWLTIENQTQSVVEDVQRIRRHPLVPSNIPIYGYLYDVRSGRLIEVPEATTAGVAR

IIIIIIIIIIIIIIIIIIIIIIIIIIIIIIIIIIIIIIIIIIIIIIIIIIIIIIIIIIIIIIIIIIIIIIIIIIIIIIIIIIIIIIIIIIIIIIIIIIIIIIIIIIIIIIIIIIIIIIIIIIII
IIIIIIIIIIIIIIIIIIIIIIIIIIIIIIIIIIIIIIIIIIIIIIIIIIIIIIIIIIIIIIIIIIII
```

>WP_159783599.1 carbonic anhydrase CahB1 [Sodalinema gerasimenkoae]

MKKLLKGLRQFKSNYFSTHQELFEQLSHGQKPRVLFITCSDSRIDPNLITNAEVGELFVIRNAGNIIPPFGAANGGEGAAVEYAVQALGV
EHIIVCGHSHCGAMKGLLKRSSLEDEMPLVYEWLRHAEATRQLLKENYSHLEGETLLEVAVAENVLTQIENLRTYPVIHSKLYRNKLHLH
AWIYHIETGEVLEYDSIRHDFVTPDENAMLEAYPVQCVLSLPEDVEELVPGHRQSSAPAQTEVAAADRDAKMPWLSSEQVARIHGGS
NGRR

Sequence
Prediction: Other

Protein type	Other	Signal Peptide (Sec/SPI)	Lipoprotein signal peptide (Sec/SPII)	TAT signal peptide (Tat/SPI)	TAT Lipoprotein signal peptide (Tat/SPII)	Pilin-like signal peptide (Sec/SPIII)
**Likelihood**	1.0001	0	0	0	0	0

Download: PNG / EPS / Tabular

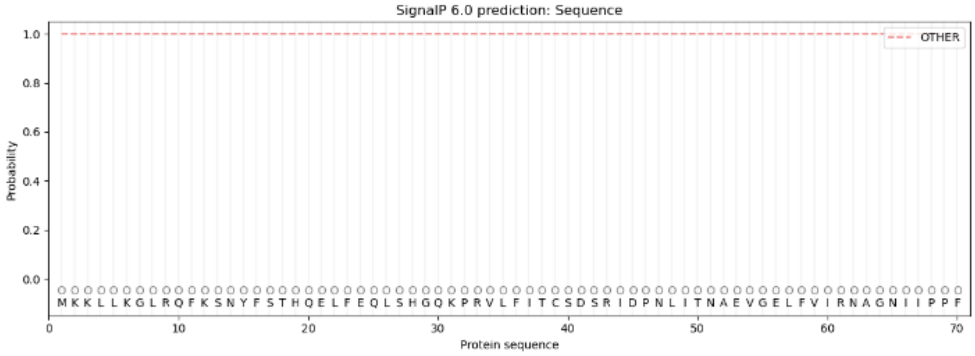

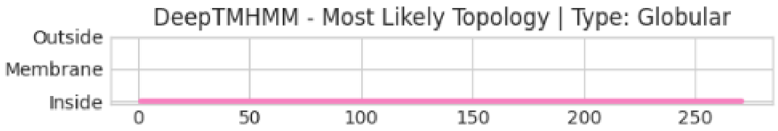

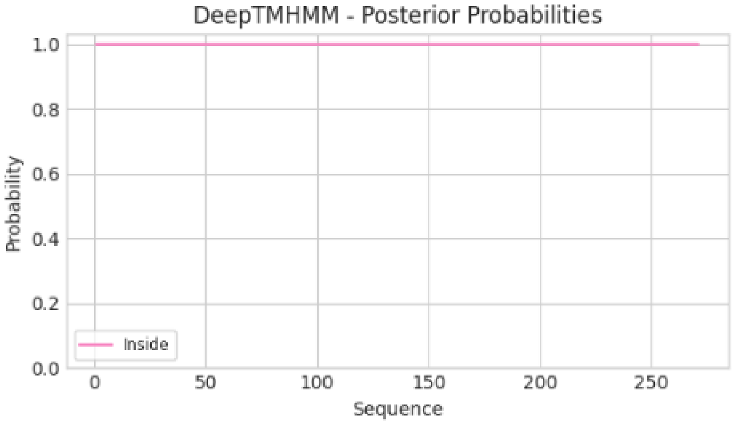


```
MKKLLKGLRQFKSNYFSTHQELFEQLSHGQKPRVLFITCSDSRIDPNLITNAEVGELFVIRNAGNIIPPFGAANGGEGAAVEYAVQALGVEHIIVCGHSHCGAMKGLLKRSSLEDEMPLVYEWL
RHAEATRQLLKENYSHLEGETLLEVAVAENVLTQIENLRTYPVIHSKLYRNKLHLHAWIYHIETGEVLEYDSIRHDFVTPDENAMLEAYPVQCVLSLPEDVEELVPGHRQSSAPAQTEVAAADR
DAKMPWLSSEQVARIHGGSNGRR

IIIIIIIIIIIIIIIIIIIIIIIIIIIIIIIIIIIIIIIIIIIIIIIIIIIIIIIIIIIIIIIIIIIIIIIIIIIIIIIIIIIIIIIIIIIIIIIIIIIIIIIIIIIIIIIIIIIIIIIIIIII
IIIIIIIIIIIIIIIIIIIIIIIIIIIIIIIIIIIIIIIIIIIIIIIIIIIIIIIIIIIIIIIIIIIIIIIIIIIIIIIIIIIIIIIIIIIIIIIIIIIIIIIIIIIIIIIIIIIIIIIIIIII
IIIIIIIIIIIIIIIIIIIIIII
```

>sp|Q54735|CYNT_SYNY3 Carbonic anhydrase CcaA OS=Synechocystis sp. (strain PCC 6803 / Kazusa) OX=1111708 GN=ccaA PE=1 SV=1

MQRLIEGLQKFREGYFSSHRDLFEQLSHGQHPRILFICCSDSRVDPNLITQSEVGDLFVIRNAGNIIPPYGAANGGEGAAMEYALVALEI
NQIIVCGHSHCGAMKGLLKLNSLQEKLPLVYDWLKHTEATRRLVLDNYSHLEGEDLIEVAVAENILTQLKNLQTYPAIHSRLHRGDLSLH
GWIYRIEEGEVLAYDGVLHDFVAPQSRINALEPEDEYAPHPNSPLISYDAFKVPGKERPGREKATESPAPQLSPLPGFGHLPREQAERIY
RGSR

Sequence
Prediction: Other

Protein type	Other	Signal Peptide (Sec/SPI)	Lipoprotein signal peptide (Sec/SPII)	TAT signal peptide (Tat/SPI)	TAT Lipoprotein signal peptide (Tat/SPII)	Pilin-like signal peptide (Sec/SPIII)
**Likelihood**	1	0	0	0	0	0

Download: PNG / EPS / Tabular

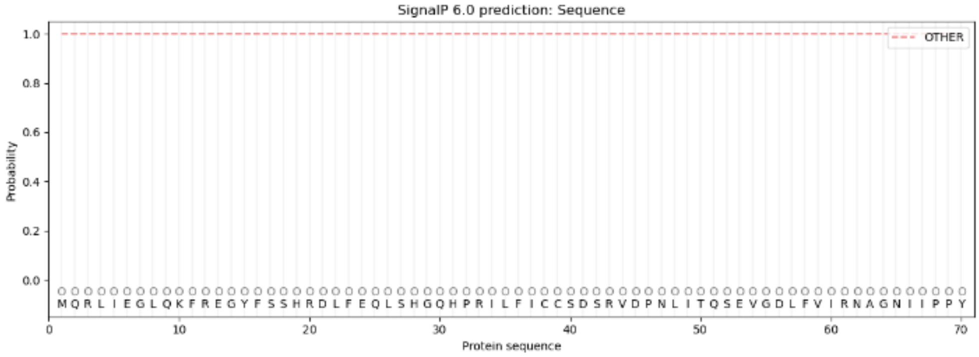

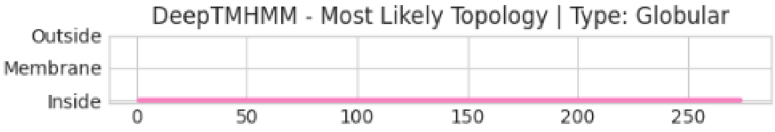

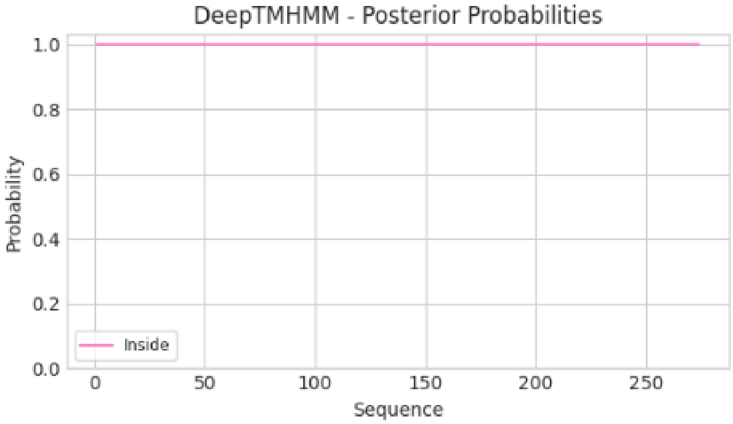


```
MQRLIEGLQKFREGYFSSHRDLFEQLSHGQHPRILFICCSDSRVDPNLITQSEVGDLFVIRNAGNIIPPYGAANGGEGAAMEYALVALEINQIIVCGHSHCGAMKGLLKLNSLQEKLPLVYDWL
KHTEATRRLVLDNYSHLEGEDLIEVAVAENILTQLKNLQTYPAIHSRLHRGDLSLHGWIYRIEEGEVLAYDGVLHDFVAPQSRINALEPEDEYAPHPNSPLISYDAFKVPGKERPGREKATESP
APQLSPLPGFGHLPREQAERIYRGSR

IIIIIIIIIIIIIIIIIIIIIIIIIIIIIIIIIIIIIIIIIIIIIIIIIIIIIIIIIIIIIIIIIIIIIIIIIIIIIIIIIIIIIIIIIIIIIIIIIIIIIIIIIIIIIIIIIIIIIIIIIIIIIII
IIIIIIIIIIIIIIIIIIIIIIIIIIIIIIIIIIIIIIIIIIIIIIIIIIIIIIIIIIIIIIIIIIIIIIIIIIIIIIIIIIIIIIIIIIIIIIIIIIIIIIIIIIIIIIIIIIIIIIIIIIIIII
IIIIIIIIIIIIIIIIIIIIIIIIII
```

>WP_011378036.1 carbonic anhydrase CcaA [Synechococcus elongatus PCC7942]

MRKLIEGLRHFRTSYYPSHRDLFEQFAKGQHPRVLFITCSDSRIDPNLITQSGMGELFVIRNAGNLIPPFGAANGGEGASIEYAIAALNIE
HVVVCGHSHCGAMKGLLKLNQLQEDMPLVYDWLQHAQATRRLVLDNYSGYETDDLVEILVAENVLTQIENLKTYPIVRSRLFQGKLQI
FGWIYEVESGEVLQISRTSSDDTGIDECPVRLPGSQEKAILGRCVVPLTEEVAVAPPEPEPVIAAVAAPPANYSSRGWLAPEQQQRIYRG
NAS

Sequence
Prediction: Other

Protein type	Other	Signal Peptide (Sec/SPI)	Lipoprotein signal peptide (Sec/SPII)	TAT signal peptide (Tat/SPI)	TAT Lipoprotein signal peptide (Tat/SPII)	Pilin-like signal peptide (Sec/SPIII)
Likelihood	1	0	0	0	0	0

Download: PNG / EPS / Tabular

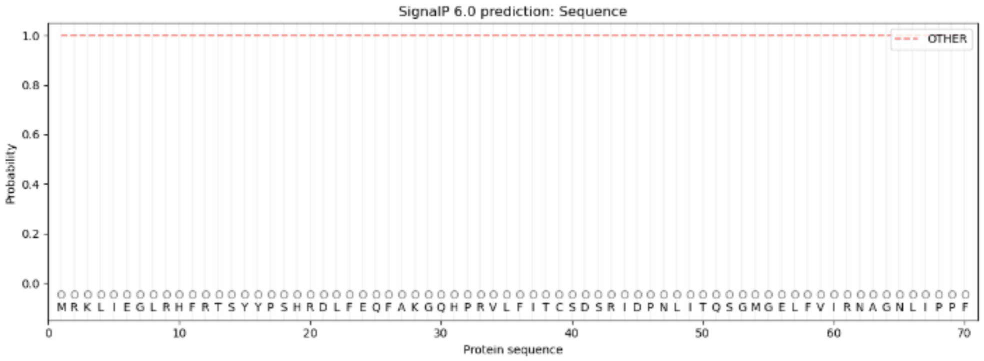

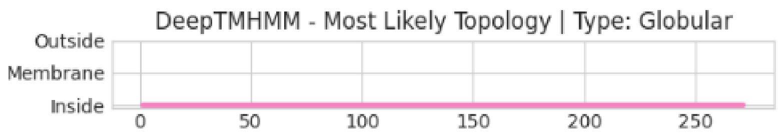

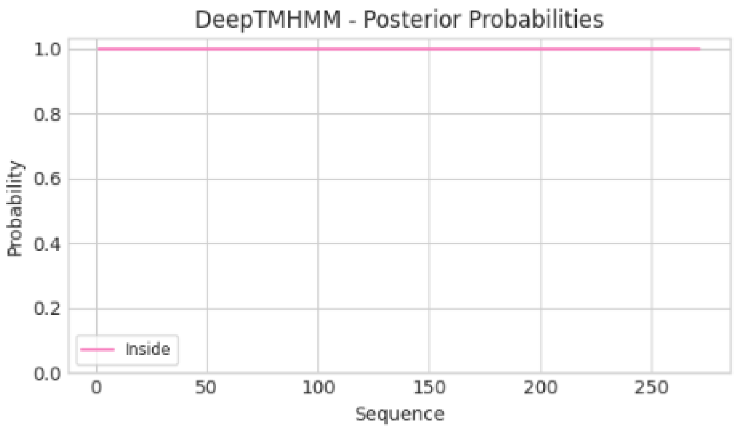


```
MRKLIEGLRHFRTSYYPSHRDLFEQFAKGQHPRVLFITCSDSRIDPNLITQSGMGELFVIRNAGNLIPPFGAANGGEGASIEYAIAALNIEHVVVCGHSHCGAMKGLLKLNQLQEDMPLVYDWL
QHAQATRRLVLDNYSGYETDDLVEILVAENVLTQIENLKTYPIVRSRLFQGKLQIFGWIYEVESGEVLQISRTSSDDTGIDECPVRLPGSQEKAILGRCVVPLTEEVAVAPPEPEPVIAAVAAP
PANYSSRGWLAPEQQQRIYRGNAS

IIIIIIIIIIIIIIIIIIIIIIIIIIIIIIIIIIIIIIIIIIIIIIIIIIIIIIIIIIIIIIIIIIIIIIIIIIIIIIIIIIIIIIIIIIIIIIIIIIIIIIIIIIIIIIIIIIIIIIIIIIII
IIIIIIIIIIIIIIIIIIIIIIIIIIIIIIIIIIIIIIIIIIIIIIIIIIIIIIIIIIIIIIIIIIIIIIIIIIIIIIIIIIIIIIIIIIIIIIIIIIIIIIIIIIIIIIIIIIIIIIIIIIII
IIIIIIIIIIIIIIIIIIIIIIII
```

>WP_012307606.1 carbonic anhydrase CcaA [Synechococcus PCC7002]

MKKLIRGLDKFKQSYVASHQDLFEQLSHGQKPRVLFICCSDSRVDPALITQTDIGEIFVIRNAGNIIPPYGAANGGEGGTLEYALQGLDIR
QIIVCGHSHCGAMKGLLKLNKLQADMPLVYDWLKHAEATRRLVRDTYPHCEGEELVETLVAENVLVQIDNLKTYPVVRSRLHQGKLKI
YGWIYNIENGEVLAYDETKHAYVKPDYSLIDETPLTEREALEGCPLPYTVASGQSLAGWYGETDTFSVSG

Sequence
Prediction: Other

Protein type	Other	Signal Peptide (Sec/SPI)	Lipoprotein signal peptide (Sec/SPII)	TAT signal peptide (Tat/SPI)	TAT Lipoprotein signal peptide (Tat/SPII)	Pilin-like signal peptide (Sec/SPIII)
Likelihood	1	0	0	0	0	0

Download: PNG / EPS / Tabular

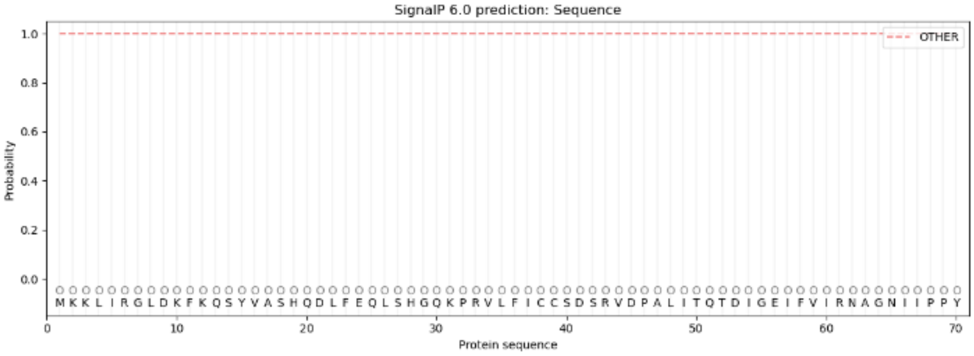

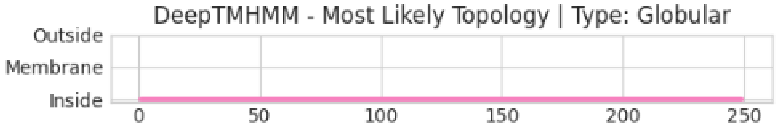

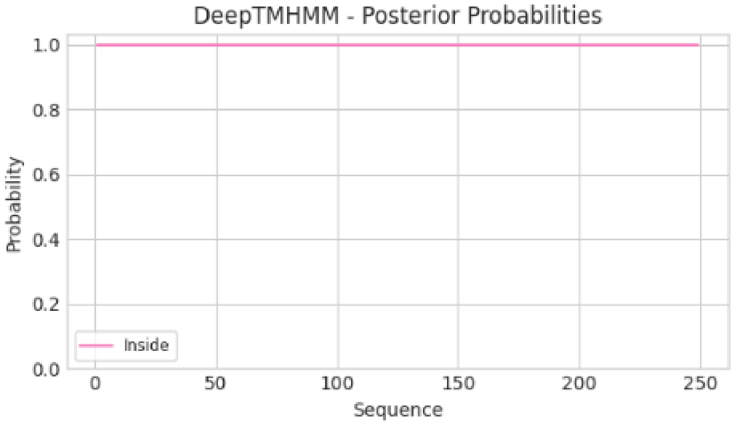


```
MKKLIRGLDKFKQSYVASHQDLFEQLSHGQKPRVLFICCSDSRVDPALITQTDIGEIFVIRNAGNIIPPYGAANGGEGGTLEYALQGLDIRQIIVCGHSHCGAMKGLLKLNKLQADMPLVYDWL
KHAEATRRLVRDTYPHCEGEELVETLVAENVLVQIDNLKTYPVVRSRLHQGKLKIYGWIYNIENGEVLAYDETKHAYVKPDYSLIDETPLTEREALEGCPLPYTVASGQSLAGWYGETDTFSVS
G

IIIIIIIIIIIIIIIIIIIIIIIIIIIIIIIIIIIIIIIIIIIIIIIIIIIIIIIIIIIIIIIIIIIIIIIIIIIIIIIIIIIIIIIIIIIIIIIIIIIIIIIIIIIIIIIIIIIIIIIIIIIII
IIIIIIIIIIIIIIIIIIIIIIIIIIIIIIIIIIIIIIIIIIIIIIIIIIIIIIIIIIIIIIIIIIIIIIIIIIIIIIIIIIIIIIIIIIIIIIIIIIIIIIIIIIIIIIIIIIIIIIIIIIII
I
```

>WP_261891106.1 carbonic anhydrase CcaA [Acaryochloris marina MBIC10699]

MKKLVRGLQEFKQSYVAQNQELLEELSHGQKPRVLFISCSDSRVDPNLITQTDVGELFVIRNAGNIVPPYGAANGGEGGTIEYAIAALEI
DQVVICGHSHCGAMKGLMKLNKLQADMPLVYDWLQHAETTRRLVAENYPESQGEERVEILVAENVLVQIDNLKTYPIVRSRLLQGKL
QIYGWIYHIETGEVLAYDDQTHTYIPPQSQLLDPPPSLPSRLEQYLISTHAPPVACEVPAPRLQSASSSPAASPVNGTPAADRIRSQLNAL
LKASPDSWVDVEDRMRSMSKLLEDARHEGMSASEAQNYHHQFSEQIPRWLRQMG

Sequence
Prediction: Other

Protein type	Other	Signal Peptide (Sec/SPI)	Lipoprotein signal peptide (Sec/SPII)	TAT signal peptide (Tat/SPI)	TAT Lipoprotein signal peptide (Tat/SPII)	Pilin-like signal peptide (Sec/SPIII)
**Likelihood**	1	0	0	0	0	0

Download: PNG / EPS / Tabular

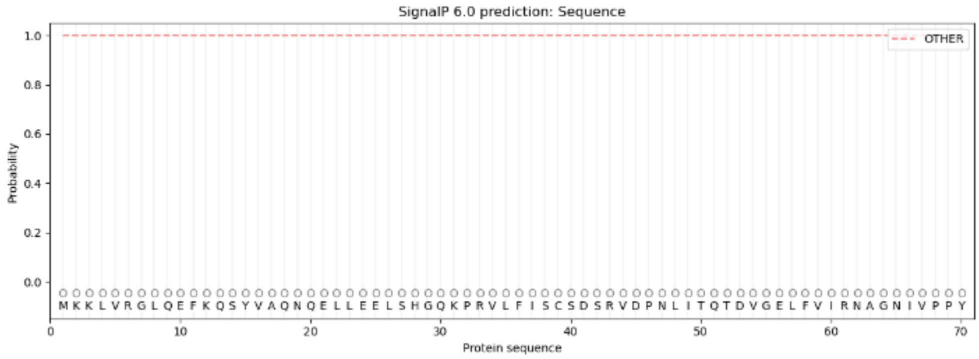

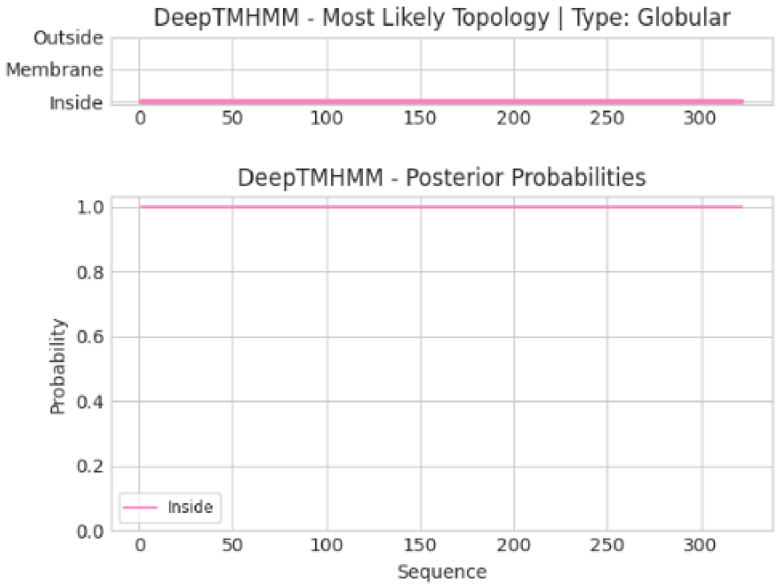

MKKLVRGLQEFKQSYVAQNQELLEELSHGQKPRVLFISCSDSRVDPNLITQTDVGELFVIRNAGNIVPPYGAANGGEGGTIEYAIAALEIDQVVICGHSHCGAMKGLMKLNKLQADMPLVYDWL
QHAETTRRLVAENYPESQGEERVEILVAENVLVQIDNLKTYPIVRSRLLQGKLQIYGWIYHIETGEVLAYDDQTHTYIPPQSQLLDPPPSLPSRLEQYLISTHAPPVACEVPAPRLQSASSSPA
ASPVNGTPAADRIRSQLNALLKASPDSWVDVEDRMRSMSKLLEDARHEGMSASEAQNYHHQFSEQIPRWLRQMG

IIIIIIIIIIIIIIIIIIIIIIIIIIIIIIIIIIIIIIIIIIIIIIIIIIIIIIIIIIIIIIIIIIIIIIIIIIIIIIIIIIIIIIIIIIIIIIIIIIIIIIIIIIIIIIIIIIIIIIIIIIII
IIIIIIIIIIIIIIIIIIIIIIIIIIIIIIIIIIIIIIIIIIIIIIIIIIIIIIIIIIIIIIIIIIIIIIIIIIIIIIIIIIIIIIIIIIIIIIIIIIIIIIIIIIIIIIIIIIIIIIIIIIII
IIIIIIIIIIIIIIIIIIIIIIIIIIIIIIIIIIIIIIIIIIIIIIIIIIIIIIIIIIIIIIIIIIIIIIIIII

>WP_012408148.1 carbonic anhydrase CcaA [Nostoc punctiforme PCC73102]

MKKLIKGLREFKSSYFSANEELFEQLSHGQKPRVLFITCSDSRIDPNLITQAGLGELFVIRNAGNIIPPFGATNGGEGATIEYAIQALDIQQII
VCGHSHCGAMKGLMKLDSLRVEMPLVHDWLKYAEATRRLVKDNYSHYEGEELLEIIIAENVLTQIENLRTYPVIRSKLHQGQLSIYAWIY
QIETGEILAYDPQKHAYVLPQNQLLSSEIDETLLSPPLNSNVEIHFTKTQQRPFQSSGELPIPTHEWFPMTVLSPEQMERIYRGSATK

Sequence
Prediction: Other

Protein type	Other	Signal Peptide (Sec/SPI)	Lipoprotein signal peptide (Sec/SPII)	TAT signal peptide (Tat/SPI)	TAT Lipoprotein signal peptide (Tat/SPII)	Pilin-like signal peptide (Sec/SPIII)
**Likelihood**	1.0001	0	0	0	0	0

Download: PNG / EPS / Tabular

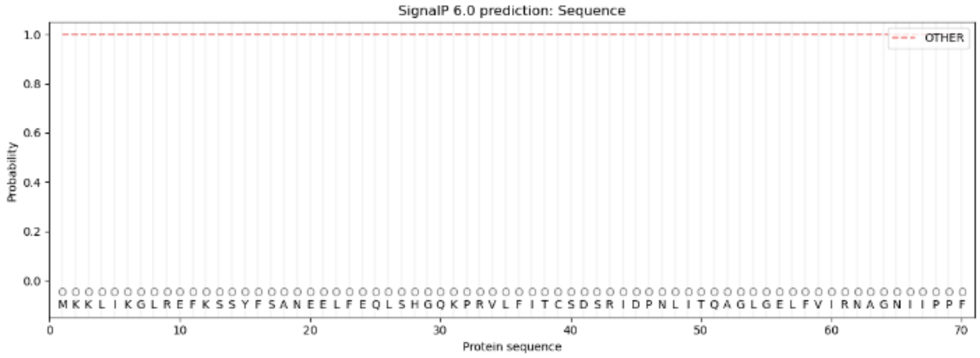

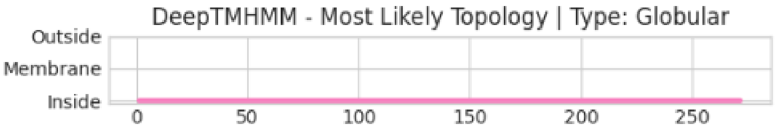

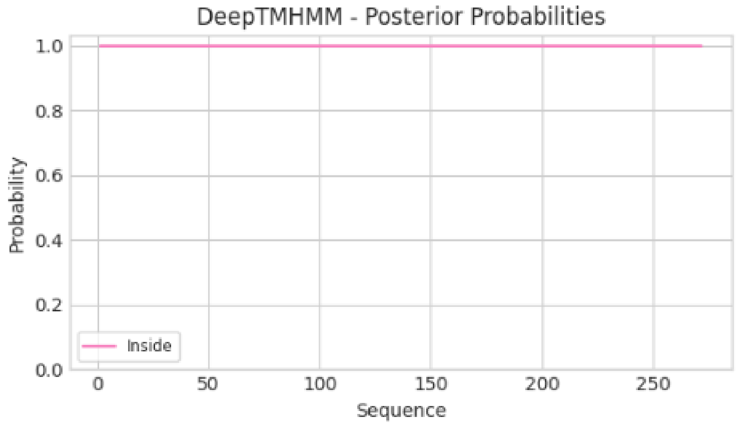


```
MKKLIKGLREFKSSYFSANEELFEQLSHGQKPRVLFITCSDSRIDPNLITQAGLGELFVIRNAGNIIPPFGATNGGEGATIEYAIQALDIQQIIVCGHSHCGAMKGLMKLDSLRVEMPLVHDWL
KYAEATRRLVKDNYSHYEGEELLEIIIAENVLTQIENLRTYPVIRSKLHQGQLSIYAWIYQIETGEILAYDPQKHAYVLPQNQLLSSEIDETLLSPPLNSNVEIHFTKTQQRPFQSSGELPIPT
HEWFPMTVLSPEQMERIYRGSATK

IIIIIIIIIIIIIIIIIIIIIIIIIIIIIIIIIIIIIIIIIIIIIIIIIIIIIIIIIIIIIIIIIIIIIIIIIIIIIIIIIIIIIIIIIIIIIIIIIIIIIIIIIIIIIIIIIIIIIIIIIIII
IIIIIIIIIIIIIIIIIIIIIIIIIIIIIIIIIIIIIIIIIIIIIIIIIIIIIIIIIIIIIIIIIIIIIIIIIIIIIIIIIIIIIIIIIIIIIIIIIIIIIIIIIIIIIIIIIIIIIIIIIIII
IIIIIIIIIIIIIIIIIIIIIIII
```

>tr|Q55136|Q55136_SYNY3 Carbonic anhydrase EcaB OS=Synechocystis sp. (strain PCC 6803 / Kazusa) OX=1111708 GN=icfA PE=3 SV=1

MMGKIEKFALTQNTSVFLVVLPDTQLNYPMAKIPTIDRRQLIQYGGAFLGTSLMATILGNQMAGNPAAQAQSANQTPQQLLTELME
GNGRFAAQKRVKANQDLYRLAEVAQGQNPFAAILSCADSRVPPEIIFDQGLGDLFICRIAGNVATPQEVGSLEFGTLVLGAKVLMVLG
HQGCGAVKAAMDGGDLPGQIGSVIKKIDIGSVTDDSSNAASVVMATKANVEHQMAVLIQSPVLSQLIAEERLLIMGAYYNLESGIVTL
L

Sequence
Prediction: Other

Protein type	Other	Signal Peptide (Sec/SPI)	Lipoprotein signal peptide (Sec/SPII)	TAT signal peptide (Tat/SPI)	TAT Lipoprotein signal peptide (Tat/SPII)	Pilin-like signal peptide (Sec/SPIII)
Likelihood	1	0	0	0	0	0

Download: PNG / EPS / Tabular

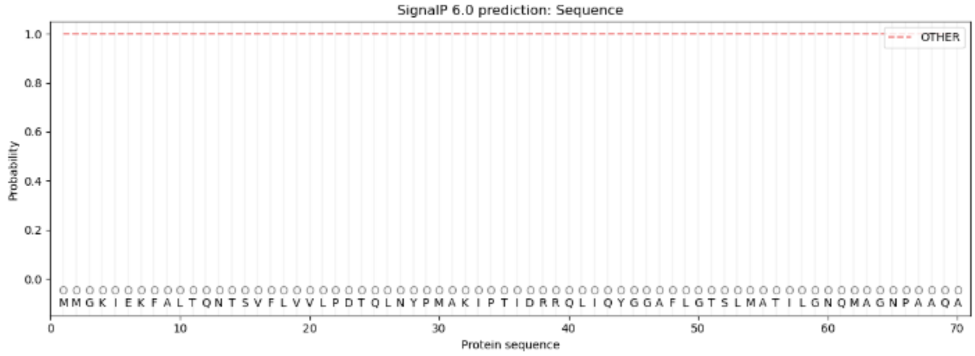

Sequence
Prediction: TAT signal peptide (Tat/SPI)

Cleavage site between pos. 41 and 42. Probability 0.828795

Protein type	Other	Signal Peptide (Sec/SPI)	Lipoprotein signal peptide (Sec/SPII)	TAT signal peptide (Tat/SPI)	TAT Lipoprotein signal peptide (Tat/SPII)	Pilin-like signal peptide (Sec/SPIII)
Likelihood	0	0	0	0.9795	0.0204	0

Download: PNG / EPS / Tabular

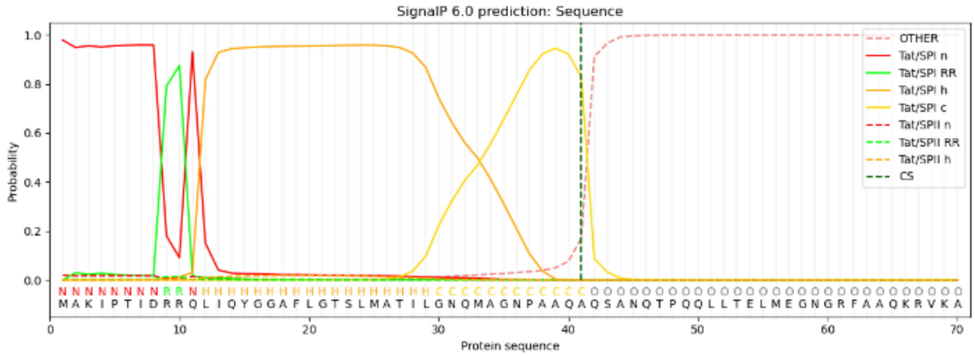

>RUR88685.1 carbonic anhydrase EcaB [Anabaena PCC 7120 = FACHB-418]

MLGKEMSRINGFIGRRNFLGLAGAGSLVVAANACAGSLTVGKEEPAIADVKSINLNPVNPQKALKLLLDGNQRFVKRKPKYPNQSRQK
LQSIAKAQYPFAAILGCADSRVPAEIVFDQGLGDLFVVRVAGNIASDMAIASLEYATSVLDTRLIVVLGHTKCGAVAASVKDEPLPGRIG
YLAESIRPALSKLPSKSKDVNKAAVIANIQYQAEKLQQKSTILAQLVNKGTVQIIGAAYDIDTGTVSVIS

Sequence
Prediction: TAT Lipoprotein signal peptide (Tat/SPII)

Cleavage site between pos. 33 and 34. Probability 0.482341

Protein type	Other	Signal Peptide (Sec/SPI)	Lipoprotein signal peptide (Sec/SPII)	TAT signal peptide (Tat/SPI)	TAT Lipoprotein signal peptide (Tat/SPII)	Pilin-like signal peptide (Sec/SPIII)
**Likelihood**	0	0	0.0001	0.3101	0.6898	0

Download: PNG / EPS / Tabular

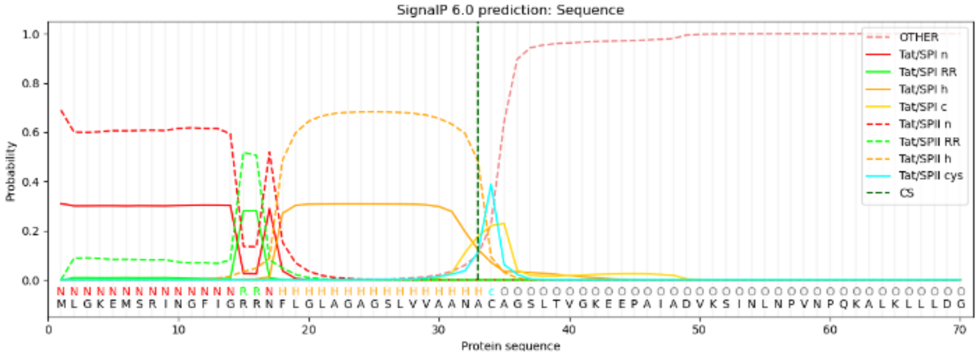

>ACC79742.1 carbonic anhydrase EcaB [Nostoc punctiforme PCC 73102]

MKHFMERRDFLKLGMTGAFGMILSASDLLWRVEQAKAADIPSTSAESLSPDAALQKLIEGNQRFVDHHPQYPDQSELRLHEVAQAQ
HPFATILSCADSRVPAEIVFDQGIGDIFDVRIAGNIATHEAIGSIEYAVVLLGSPLLMVMGHERCGAVTAAVQKESLPGDISTFVKAIKPAL
KKVKDQPGDAVENAVVANVQYQIERLQKSKLLSEQVQSGKLKIVGGRYDLDTGRVTIIT

Sequence
Prediction: TAT signal peptide (Tat/SPI)

Cleavage site between pos. 37 and 38. Probability 0.863430

Protein type	Other	Signal Peptide (Sec/SPI)	Lipoprotein signal peptide (Sec/SPII)	TAT signal peptide (Tat/SPI)	TAT Lipoprotein signal peptide (Tat/SPII)	Pilin-like signal peptide (Sec/SPIII)
**Likelihood**	0	0	0	0.9999	0.0001	0

Download: PNG / EPS / Tabular

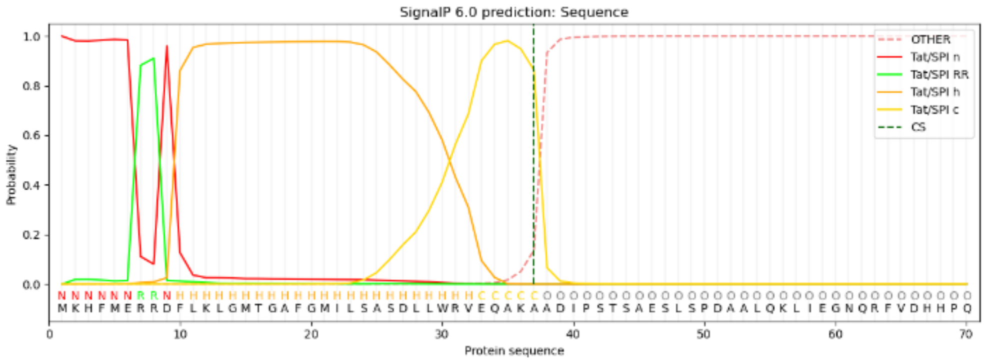

Concerning Figure 1 b:

CcaA homologues N-terminal protein alignment (aa 1-50)

```
Synechocystis sp. PCC6803 CcaA        1 MQRLIEGLQK FREGYFSSHR DLFEQLSHGQ HPRILFICCS DSRVDPNILT 50
S.gerasimenkoae IPPAS B-353 CahB1     1 MKKLLKGLRQ FKSNYFSTHQ ELFEQLSHGQ KPRVLFITCS DSRIDPNILT 50
N. punctiforme sp. PCC73102           1 MKKLIKGLRE FKSSYFSANE ELFEQLSHGQ KPRVLFITCS DSRIDPNILT 50
Synechococcus sp. PCC7002             1 MKKLIRGLDK FKQSYVASHQ DLFEQLSHGQ KPRVLFICCS DSRVDPAILT 50
A. marina MBIC10699                   1 MKKLVRGLQE FKQSYVAQNQ ELLEELSHGQ KPRVLFISCS DSRVDPNILT 50
Synechococcus sp. PCC7942             1 MRKLIEGLRH FRTSYYPSHR DLFEQFAKGQ HPRVLFITCS DSRIDPNILT 50
```
