# Supplementary material for: Extracellular CahB1 from Sodalinema gerasimenkoae IPPAS B-353 Acts as a Functional Carboxysomal β-Carbonic Anhydrase in Synechocystis sp. PCC6803
Source: Plants (Basel). 2023 Jan 6;12(2):265. doi: 10.3390/plants12020265 (PMC9865033; doi:10.3390/plants12020265)

Supplementary Materials File S4

Original Images for Blots and Gels

Concerning Figure 1 c:

Genotyping PCR gels *ccaA* locus and PpsbA2:*cahB1* expression construct amplicons. Original and unadjusted gels showing two technical replicates (top/bottom) of two biological replicates (left-right) are provided.

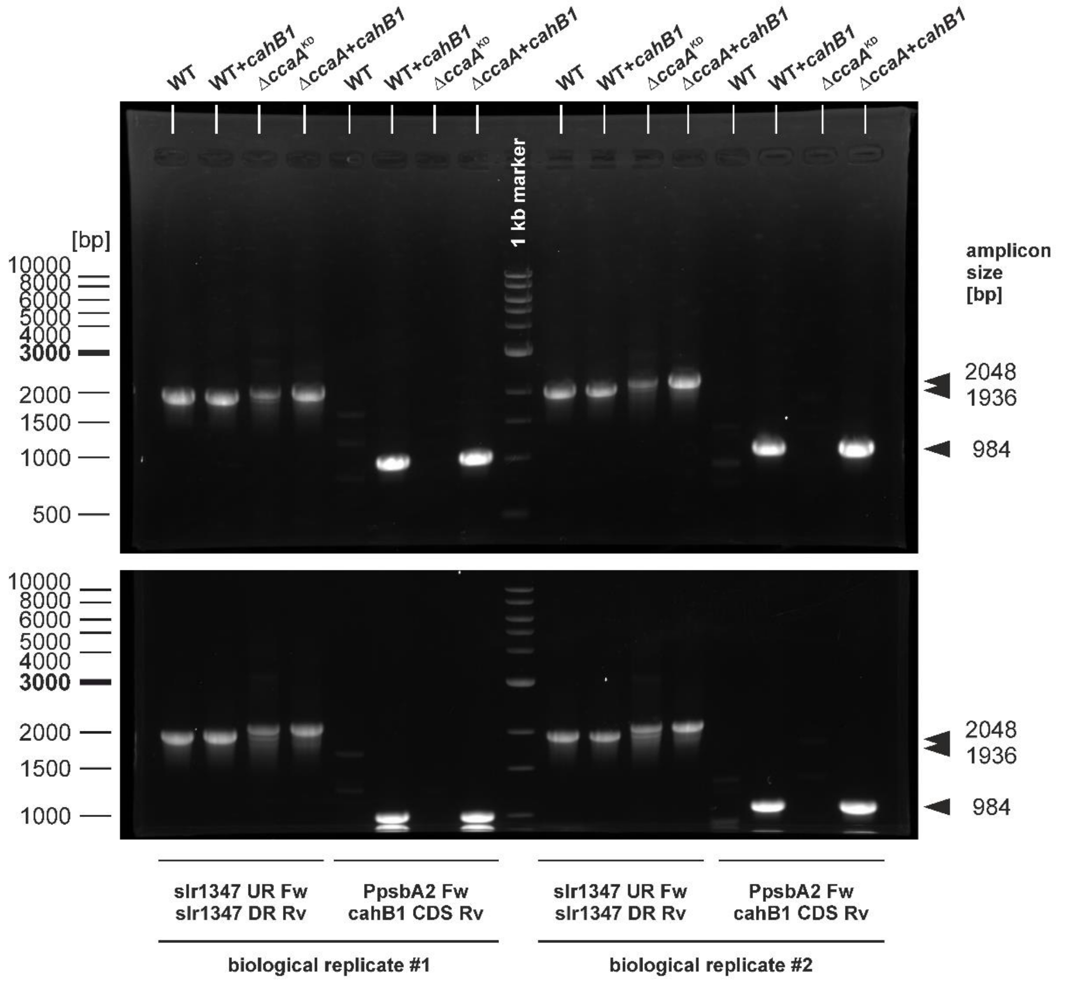

Concerning Figure 3 b/c:

Coomassie brilliant bule (CBB) stained PVDF membranes and immunoblot ECL (enhanced chemi-luminescence) detection of RbcL, CahB1, and PsbA (after re-probing the CahB1 segments of the membranes).
Original and unadjusted blot images showing results for total (T), soluble (S), pelletable (P), solubilizable (P_S) and insolubilizable (P_I) pelletable (sub-) fractions of *Synechocystis* cells. Three biological replicates of WT (top-left), WT+*cahB1* (top-right), Δ*ccaA*^{KD} (bottom left), and Δ*ccaA*+*cahB1* are provided.

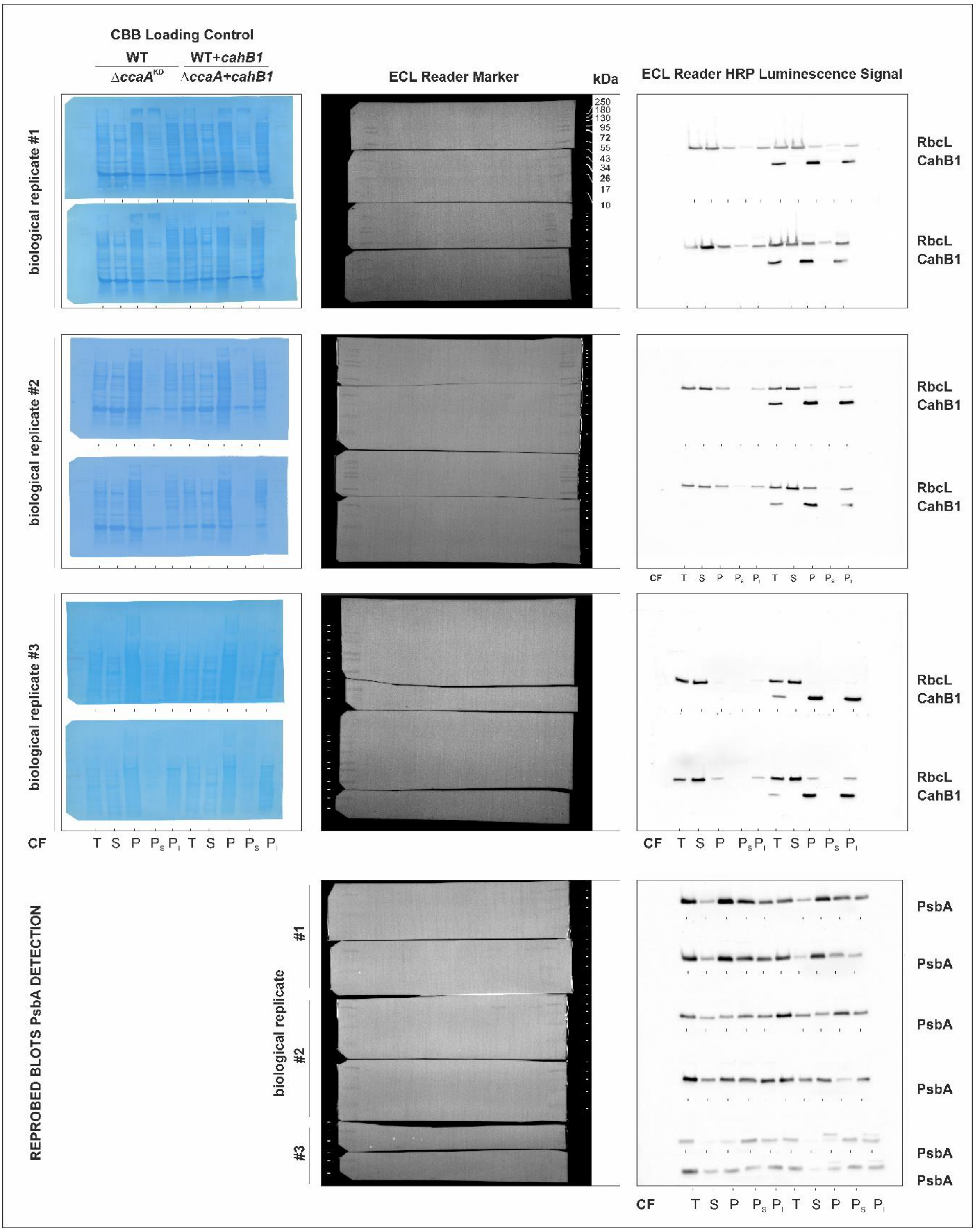

Supplement: Supplementary file 1 [file plants-12-00265-s001.zip › File S4 _ MDPI PLANTS CahB1 _ Original Images DNA Gels and Protein Blots.pdf]
